# Microbiome and cancer: mechanistic insights, diagnostic potential, and therapeutic strategies

**DOI:** 10.3389/fcell.2026.1844436

**Published:** 2026-07-03

**Authors:** Vivek Kumar, Amit Chaudhary, Mansi Gautam, Pankaj Verma, Meenu Singh

**Affiliations:** 1 USF Center for Microbiome Research, Microbiomes Institute, USF Florida, Tampa, FL, United States; 2 Center of Excellence in Aging and Brain Repair, University of South Florida Morsani College of Medicine, Tampa, FL, United States; 3 Department of Cell, Developmental and Integrative Biology, Heersink School of Medicine, The University of Alabama at Birmingham, Birmingham, AL, United States; 4 Department of Pathology, Sharda University, Greater Noida, Uttar Pradesh, India; 5 Department of Blood, Sharda University, Greater Noida, Uttar Pradesh, India; 6 Department of Biotechnology, IILM University, Greater Noida, Uttar Pradesh, India

**Keywords:** cancer diagnostics, immune modulation, microbial metabolites, microbiome–cancer interaction, therapeutic response

## Abstract

The human microbiome is now recognized as an active and dynamic participant in cancer biology rather than a passive bystander. Increasing evidence demonstrates that microbial dysbiosis contributes to tumor initiation and progression through chronic inflammation, genotoxic toxin production, metabolic reprogramming, immune modulation, and direct reshaping of the tumor microenvironment. Specific microbial factors including colibactin, *Bacteroides fragilis* toxin, CagA, and *Fusobacterium* adhesins intersect with canonical oncogenic pathways. Linking microbial activity to genomic instability and immune evasion. Microbial metabolites such as secondary bile acids, lipopolysaccharide, hydrogen sulfide, and short-chain fatty acids further regulate epithelial integrity, epigenetic remodeling, and immune cell dynamics in a context-dependent manner. Beyond tumorigenesis, the microbiome critically determines therapeutic response. Microbial communities influence chemotherapy and radiotherapy outcomes and shape immune checkpoint blockade efficacy through immune priming, antigen mimicry, and microbiome–metabolite–immune interactions that govern treatment responsiveness. Emerging preclinical studies and early clinical investigations suggest that microbiome modulation, including fecal microbiota transplantation (FMT), may help restore immunotherapy sensitivity in selected patients; however, larger controlled trials are required to establish efficacy, safety, and long-term clinical benefits. This review integrates mechanistic, preclinical, and clinical evidence across microbiome-driven carcinogenesis, tumor microenvironment remodeling, drug metabolism, and biomarker development. Advances in circulating microbial DNA profiling and machine learning–based diagnostics further position the microbiome as both a mechanistic driver and a translational target in precision oncology. We also discuss key challenges, including interindividual variability, standardization of methodologies, and the need for personalized therapeutic strategies. Collectively, understanding and harnessing microbiome–cancer interactions hold significant promise for improving cancer diagnosis, treatment, and patient outcomes.

## 1 Introduction

Cancer is one of the leading causes of mortality worldwide, with its progression driven by a complex interplay of genetic, environmental, immune, and microbial factors. While genetic mutations and lifestyle factors have been extensively studied, the role of microbiome in cancer development and treatment response has gained increasing attention in recent years (Cullin et al., 2021). The human microbiome, composed of bacteria, viruses, fungi, archaea, and their metabolites, plays a crucial role in maintaining homeostasis. However, microbial dysbiosis (an imbalance in microbial communities) has been implicated in carcinogenesis by inducing chronic inflammation, producing genotoxic metabolites, and altering immune responses (Ayivi-Tosuh et al., 2026).

Recent advances in next-generation sequencing (NGS) and metagenomic analysis have revealed the diverse interactions between host microbiota and tumor biology, leading to the identification of microbial biomarkers and therapeutic targets (Che et al., 2024; Kumar, et al., 2024a; Kumar, et al., 2024b). For instance, studies have identified tumor-associated microbiota that influences the tumor microenvironment (TME) and affect immune evasion, drug resistance, and metastasis (Guo et al., 2024; He et al., 2022). Specific bacteria such as *Helicobacter pylori (H. pylori)* (gastric cancer), *Fusobacterium nucleatum (F. nucleatum)* (colorectal cancer), and colibactin-producing *Escherichia coli* have been implicated in promoting tumor growth through DNA damage, pro-inflammatory cytokine release, and immunosuppressive mechanisms (de Oliveira Alves et al., 2024; Risoen et al., 2025; Salvatori et al., 2023).

Beyond tumorigenesis, the microbiome also plays a significant role in modulating cancer treatment responses. Studies show that gut microbiota composition can influence the efficacy of chemotherapy, radiotherapy, and immunotherapy, with certain bacterial species enhancing or inhibiting therapeutic outcomes (Cheng et al., 2020; Lei et al., 2025). For example, gut microbiota composition is a critical determinant of response to immune checkpoint inhibitors (ICIs) such as anti-PD-1/PD-L1 therapy, with beneficial bacteria (*A*. *muciniphila* and, *Bifidobacterium*) enhancing anti-tumor immunity (Lu et al., 2025; Wang et al., 2020). Conversely, antibiotic use can disrupt gut microbiota, reduce treatment efficacy and increase resistance (Mafe and Büsselberg, 2025; Miller et al., 2025).

**FIGURE 1:**
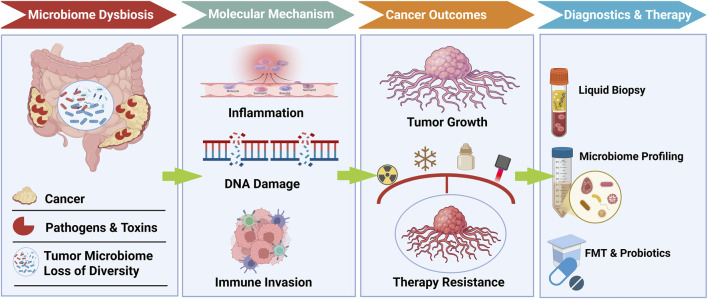
Conceptual framework linking microbiome dysbiosis to cancer initiation, progression, and clinical translation. Microbiome dysbiosis, characterized by loss of microbial diversity and enrichment of pathogenic microbes, contributes to cancer development through multiple interconnected mechanisms. Dysbiotic microbial communities produce genotoxic toxins and metabolites that induce DNA damage, chronic inflammation, and immune invasion, thereby reshaping the tumor microenvironment. These processes promote tumor growth, immune evasion, and resistance to anticancer therapies. Advances in microbiome research have enabled the translation of these insights into clinical applications, including microbiome-based diagnostics such as liquid biopsy and microbial profiling, as well as therapeutic strategies such as fecal microbiota transplantation (FMT), probiotics, and microbiome modulation to enhance treatment efficacy. This figure provides an overview of the microbiome–cancer axis, serving as a roadmap for the mechanistic and translational concepts discussed throughout the review.

Given these insights, microbiome-targeted therapeutic strategies are being explored to enhance cancer treatment outcomes. Approaches such as probiotic and prebiotic supplementation, fecal microbiota transplantation (FMT), dietary interventions, and microbiome engineering have shown promising results in preclinical studies and selected early-phase clinical trials; however, their efficacy remains variable and requires further validation in larger patient cohorts (Cho et al., 2024; Kim et al., 2025). However, translating microbiome-based strategies into clinical practice remains a challenge due to interindividual microbiome variability, lack of standardized microbiome profiling methods, and ethical considerations (Tegegne and Savidge, 2025). The framework linking microbiome dysbiosis to cancer initiation, progression, and clinical translation are summarized in Figure 1.

This review provides a comprehensive analysis of microbiome-cancer interactions, covering the mechanisms of microbial involvement in tumorigenesis, microbiome-based diagnostic biomarkers, and therapeutic strategies. Specifically, we discuss the role of microbial dysbiosis in cancer development through inflammation, immune suppression, and metabolite-driven oncogenesis. The microbiome’s influence on cancer therapies, including chemotherapy, radiotherapy, and immunotherapy, is explored in detail. Furthermore, we evaluate microbiome-based cancer diagnostic and therapeutic strategies, including probiotics, prebiotics, and fecal microbiota transplantation. Finally, we highlight challenges and future directions in microbiome oncology, emphasizing personalized medicine and AI-driven microbiome analysis. By integrating microbiome research with precision oncology, this review aims to highlight emerging trends and future directions in microbiome-based cancer treatment. Understanding the microbiome’s role in cancer will pave the way for personalized therapeutic approaches, ultimately improving patient outcomes in oncology.

## 2 Microbiome in cancer development and progression

The human microbiome plays an essential role in regulating immune responses, maintaining epithelial integrity, and metabolizing various compounds (Cortés et al., 2025). However, microbial dysbiosis an imbalance in microbial composition, has been increasingly recognized as a key contributor to cancer development and progression. Dysbiosis leads to chronic inflammation, DNA damage, and immune evasion, all of which are established hallmarks of cancer (Zhao et al., 2023). Several bacterial species, viruses, and fungal organisms have been implicated in tumorigenesis, either through direct mutagenic effects or by modulating the host’s immune landscape. In addition, microbial metabolites influence key oncogenic pathways, making the microbiome a crucial player in cancer progression (Chen et al., 2026).

### 2.1 Microbial dysbiosis and oncogenesis

One of the most well-established microbial carcinogens is *Helicobacter pylori (H*. *pylori)*, a Gram-negative bacterium that colonizes the gastric mucosa and is classified as a Group 1 carcinogen by the International Agency for Research on Cancer (IARC). *H*. *pylori* induces chronic inflammation, disrupts the gastric epithelial barrier, and promotes genetic instability, increasing the risk of gastric adenocarcinoma (Nomura et al., 1991; Reyes, 2023). The bacterium injects cytotoxin-associated gene A (CagA) into host cells via a Type IV secretion system, where it interacts with Wnt/β-catenin and p53 pathways, leading to uncontrolled cellular proliferation. Another virulence factor, vacuolating cytotoxin A (VacA), induces apoptosis and increases oxidative stress, further promoting DNA damage and tumorigenesis (Takahashi-Kanemitsu et al., 2020). In colorectal cancer (CRC), *F*. *nucleatum* has emerged as a key microbial contributor to tumorigenesis. This anaerobic bacterium is normally found in the oral cavity, but it can migrate to the gut, where it promotes tumor progression. Studies have identified *F. nucleatum* enrichment in CRC tissues, where it interacts with tumor cells through FadA adhesin, binding to E-cadherin and activating β-catenin signaling, which enhances cell proliferation and invasion (Galasso et al., 2025; Kowsarkhizi et al., 2025). In addition, *F. nucleatum* creates a pro-tumorigenic microenvironment by inducing myeloid-derived suppressor cells (MDSCs) and suppressing tumor-infiltrating T cells, effectively evading immune surveillance (Kostic et al., 2013). Another major contributor to colorectal carcinogenesis is colibactin-producing *Escherichia coli* (*E. coli*). Specific strains of *E. coli* harbor the polyketide synthase (pks) genomic island, which encodes colibactin, a genotoxin that induces double-strand DNA breaks and genomic instability (Sadeghi et al., 2024). Colibactin-induced DNA damage has been shown to create mutational signatures in CRC patients, confirming its role in colorectal tumorigenesis (Dziubańska-Kusibab et al., 2020; Pleguezuelos-Manzano et al., 2020).

Viruses and fungi are also implicated in cancer development. *Human papillomavirus* (HPV), Epstein-Barr virus (EBV), and hepatitis B and C viruses (HBV/HCV) are well-documented oncogenic viruses that contribute to cervical, gastric, and hepatocellular carcinomas, respectively (McLaughlin-Drubin and Munger, 2008). Meanwhile, fungal species such as *Malassezia* and *Candida* have been linked to pancreatic and colorectal cancers, largely through their ability to induce chronic inflammation and immune suppression (Aykut et al., 2019).

### 2.2 Microbial metabolites and tumorigenesis

Microbial metabolites play a crucial role in carcinogenesis by influencing key pathways involved in genomic instability, immune modulation, and chronic inflammation. These metabolites, produced by gut bacteria, fungi, and viruses, can either promote or suppress tumor growth, depending on their biochemical properties and interactions with host cells. Some bacterial species generate genotoxic compounds that induce DNA damage, while others produce immunomodulatory metabolites that impact the tumor microenvironment (Qu et al., 2023; Rajak et al., 2026).

A well-characterized class of microbial carcinogens includes secondary bile acids, such as deoxycholic acid (DCA) and lithocholic acid (LCA), which are generated by gut commensal bacteria through the metabolism of primary bile acids. Elevated levels of DCA have been strongly associated with colorectal cancer and hepatocellular carcinoma, where DCA promotes tumor progression by inducing reactive oxygen species, oxidative DNA damage, and epithelial–mesenchymal transition. Recent evidence further indicates that DCA enhances angiogenic signaling during intestinal carcinogenesis via activation of vascular endothelial growth factor receptor-2 (VEGFR2), thereby supporting tumor vascularization and invasive potential (Song et al., 2022; Yang and Qian, 2022). Another major microbial metabolite involved in oncogenesis is colibactin, a genotoxin produced by pks + *E*. *coli*. Colibactin has been shown to alkylate DNA, induce double-strand breaks, and promote chromosomal instability, leading to mutagenesis in colorectal epithelial cells (Dalmasso et al., 2014). In addition to metabolite-mediated genotoxicity, certain pathogenic bacteria such as *Salmonella enterica (S. enterica)* promote DNA damage indirectly by activating host signaling pathways, including PI3K-Akt, which compromises genomic stability and enhances tumorigenic potential in colonic epithelial cells (Iftekhar et al., 2021).

In contrast to genotoxic and pro-inflammatory microbial metabolites, particularly SCFAs, butyrate, acetate, and propionate, represent an important class of microbiota-derived metabolites with context-dependent roles in tumorigenesis. These metabolites are generated through the fermentation of dietary fibers by gut commensals such as *Bacteroides*, *Clostridium*, and *Faecalibacterium* species. Among SCFAs, butyrate has been extensively studied for its role in maintaining colonic epithelial homeostasis. It functions as a histone deacetylase inhibitor, thereby modulating gene expression programs that promote cellular differentiation, limit inflammation, and suppress tumor growth in the colon (Liu et al., 2018; Son and Cho, 2023). The biological effects of butyrate are strongly influenced by cellular metabolic state, a phenomenon commonly referred to as the “butyrate paradox.” In normal colonocytes, butyrate serves as a primary oxidative energy source and supports epithelial renewal. In contrast, colorectal cancer cells exhibit altered metabolic programming characterized by increased reliance on aerobic glycolysis, which limits butyrate oxidation. As a result, butyrate accumulates intracellularly and exerts enhanced epigenetic effects through histone deacetylase inhibition, leading to growth arrest and reduced proliferation of tumor cells. This metabolic selectivity underlies the tumor-suppressive properties of butyrate in colorectal cancer and highlights its potential relevance in cancer prevention and therapeutic modulation (Yamamoto and Sakisaka, 2012). Lipopolysaccharides (LPS), components of the outer membranes of Gram-negative bacteria, are another class of microbial metabolites that modulate tumor progression. LPS activates Toll-like receptor 4 (TLR4) signaling, which triggers an inflammatory cascade involving NF-κB, IL-6, and TNF-α, all of which promote tumor cell proliferation and survival (Zhang et al., 2021; Zu et al., 2017). LPS-induced inflammation is particularly significant in colorectal, liver, and pancreatic cancers, where chronic exposure to bacterial endotoxins fuels tumor progression through immune suppression and angiogenesis enhancement (Li et al., 2023; Sivam et al., 2023; Wang et al., 2019).

Additionally, hydrogen sulfide (H_2_S), produced by sulfate-reducing bacteria like *Desulfovibrio* and *Bilophila wadsworthia*, has been implicated in colorectal cancer progression. H_2_S influences tumor metabolism, oxidative stress, and mitochondrial function, thereby enhancing cancer cell proliferation while also inhibiting apoptosis in colorectal epithelial cells (Lin et al., 2023). Beyond classical microbial metabolites, the gut microbiome also regulates host hormone metabolism, thereby influencing systemic cancer risk. Certain bacterial taxa, including *E*. *coli* and *Enterobacter* species, express β-glucuronidase enzymes that deconjugate glucuronidated estrogens and androgens in the intestine, enabling their reactivation and recirculation in bioactive forms. Dysregulation of this microbial hormone metabolism has been implicated in hormone-dependent malignancies, such as breast and prostate cancer, through increased systemic exposure to estrogens and androgens (Ervin et al., 2019; Sui et al., 2021).

Taken together, microbial metabolites act as central regulators of tumorigenesis by influencing genomic stability, immune surveillance, inflammatory signaling, and host metabolic pathways. While certain metabolites, such as short-chain fatty acids, contribute to epithelial homeostasis and epigenetic regulation, others including secondary bile acids, microbial genotoxins, and lipopolysaccharides promote DNA damage, chronic inflammation, immune suppression, and tumor progression. These context-dependent effects highlight the dualistic nature of microbiome-derived metabolites in cancer biology. Importantly, the clinical relevance of these metabolites extends beyond mechanistic tumorigenesis. Metabolite signatures, including SCFAs, secondary bile acids, and LPS-associated inflammatory profiles, may serve as potential biomarkers for cancer risk assessment, disease progression, treatment response monitoring, and patient stratification.

**FIGURE 2:**
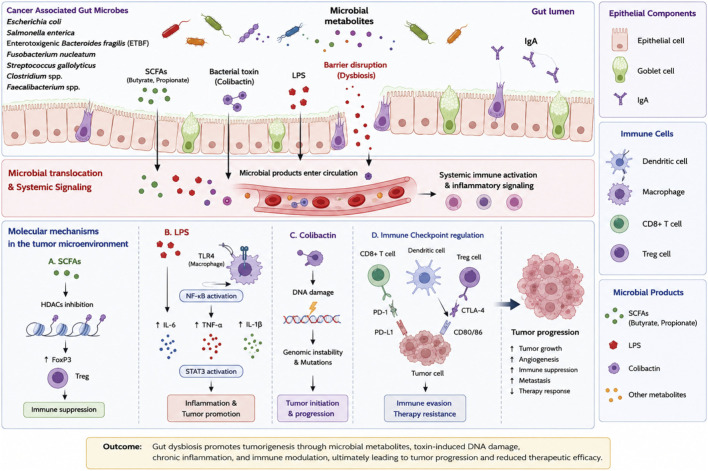
Integrated mechanisms by which cancer-associated gut microbes and microbial metabolites drive tumorigenesis and immune modulation across the gut–tumor axis. Cancer-associated gut dysbiosis leads to enrichment of pathogenic and metabolically active microbes, including *Escherichia coli*, *Salmonella enterica*, *Streptococcus gallolyticus*, enterotoxigenic *Bacteroides fragilis* (ETBF), *Clostridium*, and *Faecalibacterium*. These microbes produce genotoxins, inflammatory ligands, and immunomodulatory metabolites that disrupt intestinal barrier integrity and facilitate microbial translocation. Microbial products such as colibactin, LPS, and SCFAs gain access to the lamina propria and systemic circulation, exerting distal effects within the tumor microenvironment. Colibactin induces DNA double-strand breaks and genomic instability in epithelial and tumor cells, promoting tumor initiation and progression. LPS activates macrophage TLR4–MD2–CD14 signaling, leading to NF-κB–dependent cytokine production (IL-6, TNF-α, IL-1β) and downstream STAT3 activation, thereby driving chronic inflammation, angiogenesis, immune suppression, and myeloid-derived suppressor cell expansion. In parallel, context-dependent SCFA signaling (butyrate and propionate) through GPR43/GPR109A and histone deacetylase inhibition promotes epigenetic remodeling and FoxP3^+^ regulatory T-cell expansion with increased CTLA-4 expression. These convergent pathways suppress CD8^+^ T-cell cytotoxicity, reinforce immune checkpoint dominance, and facilitate tumor immune evasion and progression. Collectively, this figure illustrates how microbial dysbiosis and metabolite-driven mechanisms link the gut microbiome to tumor microenvironment remodeling.

Integration of metabolomic and microbiome profiling may facilitate the development of personalized microbiome-targeted interventions and precision oncology strategies. A deeper understanding of these complex host–microbe interactions therefore provides opportunities for biomarker discovery, therapeutic monitoring, and microbiome-directed therapies, including dietary modulation, probiotic or prebiotic interventions, and inhibition of tumor-promoting microbial pathways. The integrated mechanisms by which cancer-associated gut microbes and their metabolites drive DNA damage, inflammatory cascades, epigenetic remodeling, and immune evasion along the gut–tumor axis are summarized in Figure 2.

### 2.3 Microbiome–epigenetic interactions in cancer

The microbiome exerts profound effects on host gene regulation through epigenetic mechanisms, including DNA methylation, histone modification, and chromatin remodeling. These changes are dynamic and reversible, enabling microbial signals to influence transcriptional programs in response to environmental and metabolic cues, thereby linking host-microbe interactions to cancer initiation and progression (Kumar et al., 2019; 2026).

A key interface between the microbiome and epigenetic regulation is mediated by microbial metabolites. Short-chain fatty acids (SCFAs), particularly butyrate, propionate, and acetate, are produced by commensal bacteria such as *Faecalibacterium prausnitzii (F. prausnitzii)* and *Roseburia* spp., which are major contributors to colonic butyrate production (Louis and Flint, 2017). These metabolites can function as histone deacetylase inhibitors, leading to increased histone acetylation and altered gene expression, thereby influencing cancer cell proliferation and survival (Donohoe et al., 2012). This establishes a direct mechanistic link between microbial composition and host epigenetic regulation in cancer.

Beyond histone regulation, the microbiome also influences DNA methylation through modulation of one-carbon metabolism. Gut microbes regulate folate availability and S-adenosylmethionine (SAM) synthesis, the universal methyl donor required for DNA methyltransferase (DNMT)-mediated methylation. Dysbiosis can disrupt this metabolic equilibrium, leading to aberrant DNA methylation patterns, including tumor suppressor gene silencing and genomic instability (Zhao et al., 2021).

Importantly, emerging evidence links specific microbial taxa to host DNA methylation alterations in cancer. *F*. *nucleatum* has been associated with CpG island methylator phenotype–related colorectal cancer features, particularly in the serrated pathway (Ito et al., 2015). In contrast, *H*. *pylori* provides stronger mechanistic evidence of microbe-driven epigenetic remodeling, inducing chronic inflammation and an epigenetic field defect that predisposes to gastric cancer (Ushijima and Hattori, 2012).

In addition to metabolite-mediated regulation, microbial-derived inflammatory signals such as lipopolysaccharide (LPS) from Gram-negative bacteria can reshape the host epigenetic landscape by inducing DNA methylation changes. LPS has been shown to alter DNA methylation profiles and gene expression in epithelial and cancer cells (Permain et al., 2025). These effects are mediated in part through activation of NF-κB signaling, which can recruit DNA methyltransferases to chromatin and promote aberrant promoter methylation in cancer (Liu et al., 2012). In parallel, inflammatory signaling pathways, including STAT3, have been implicated in regulating DNMT expression and reinforcing epigenetic silencing programs (Zhang et al., 2006). Together, these findings link microbiome-driven inflammatory signaling to epigenetic dysregulation in tumorigenesis.

Emerging evidence further indicates that microbiome-driven epigenetic regulation plays a critical role in therapeutic response. By modulating DNA methylation and histone modifications, the microbiome can influence immune checkpoint gene expression, antigen presentation, and T-cell activation, thereby impacting responsiveness to immune checkpoint blockade (Gopalakrishnan et al., 2018; Routy et al., 2018). Microbiome-dependent immune priming and metabolite-driven signaling further shape antitumor immunity and treatment outcomes (Zhou et al., 2019). These findings support a microbiome–metabolite–epigenetic–immune axis integrating metabolic and immune pathways in cancer progression and therapy response.

Collectively, these findings establish microbial dysbiosis, metabolite signaling, and epigenetic remodeling as interconnected mechanisms linking the microbiome to tumor initiation, progression, and therapeutic responsiveness. Importantly, microbiome-associated epigenetic alterations may provide clinically actionable biomarkers for cancer risk assessment, early detection, therapeutic monitoring, and patient stratification. Identification of microbiome-driven DNA methylation and chromatin remodeling signatures could facilitate precision oncology approaches by enabling prediction of treatment response and guiding microbiome-targeted therapeutic interventions. Beyond these oncogenic and regulatory processes, the microbiome also plays a central role in shaping host immunity. Building upon the mechanisms discussed above, the following section focuses on how microbiota influences antitumor immune responses, immune checkpoint signaling, and cancer immunotherapy outcomes.

## 3 Molecular mechanisms of microbiome-cancer interactions

Building upon the oncogenic, metabolic, and epigenetic mechanisms discussed in Section 2, accumulating evidence indicates that the microbiome also directly influences antitumor immunity and therapeutic responsiveness. This section focuses on immune-mediated mechanisms, including inflammatory signaling, immune-cell regulation, immune checkpoint modulation, and microbial antigen mimicry, that shape cancer progression and treatment outcomes.

### 3.1 Microbial influence on the immune system: pro-inflammatory microbes and tumorigenesis

As discussed in Section 2, chronic inflammation represents a major mechanism linking microbial dysbiosis to tumor development. Here, we focus specifically on how microbiota-driven inflammatory signaling reshapes the tumor immune microenvironment and influences cancer progression. Persistent inflammatory responses, often mediated by microbial components such as LPS, tumor necrosis factor-alpha (TNF-α), and interleukin-6 (IL-6), create a pro-tumorigenic microenvironment. Chronic NF-κB activation, a consequence of microbial dysbiosis, drives oncogenic pathways by enhancing cell proliferation and inhibiting apoptosis (Araújo-Filho, 2024; Francescone et al., 2014; Ivleva and Grivennikov, 2022). Certain bacterial species, such as *F*. *nucleatum*, promote inflammation-driven tumorigenesis by recruiting myeloid-derived suppressor cells (MDSCs) and regulatory T cells (Tregs), both of which suppress anti-tumor immune responses (Li et al., 2025; Zhang et al., 2026). *F. nucleatum* has been shown to stimulate IL-8 and TNF-α secretion from colonic epithelial cells, fueling a chronic inflammatory state that facilitates tumor growth. Additionally, *H*. *pylori* contribute to gastric cancer progression by activating NF-κB and TLR signaling pathways, leading to cytokine production and immune cell infiltration into the tumor microenvironment (Engevik et al., 2021; Galasso et al., 2025). In colorectal cancer, dysbiotic gut microbiota enriches the tumor microenvironment with LPS-producing bacteria, which stimulate TLR4 signaling, inducing pro-inflammatory cytokines such as IL-6, IL-1β, and TNF-α (Li et al., 2023). This chronic inflammatory signaling contributes to epithelial-mesenchymal transition (EMT), which enhances tumor invasiveness and metastasis (Figure 2).

### 3.2 Microbiome and immune checkpoints

A growing body of preclinical evidence has established a direct, causal relationship between gut microbiota composition and therapeutic responses to immune checkpoint inhibitors (ICIs). Studies using murine tumor models have demonstrated that both defined microbial consortia and individual bacterial species can significantly enhance antitumor immunity in response to PD-1/PD-L1 and CTLA-4 blockade. Mechanistically, microbiome-mediated enhancement of ICI efficacy can be broadly categorized into four interconnected pathways: (i) enhancement of dendritic cell activation and antigen presentation, (ii) promotion of effector T-cell priming and Th1-polarized immunity, (iii) microbial metabolite-mediated immune modulation, and (iv) antigen mimicry-driven activation of tumor-reactive immune responses.

#### 3.2.1 Enhancement of dendritic cell activation and antigen presentation

One of the most consistently reported mechanisms through which the microbiome enhances immune checkpoint efficacy is the stimulation of dendritic cell maturation and antigen presentation. Several investigations have employed defined bacterial consortia to restore or enhance responsiveness to PD-1/PD-L1 inhibition. These include consortia composed predominantly of *Bifidobacterium* species, mixtures enriched in *Clostridiales*, and an eleven-strain consortium comprising *Parabacteroides*, *Alistipes*, *Paraprevotella*, *Bacteroides*, *Eubacterium*, *Clostridiales*, *Phascolarctobacterium*, and *Fusobacterium*. Collectively, these microbial communities promote dendritic cell activation, enhance antigen presentation, improve T-cell priming, and strengthen tumor-specific immune responses (Hwang et al., 2023; Lei et al., 2025; Pamungkas et al., 2025).

Similarly, individual bacterial strains have been shown to be sufficient to enhance ICI efficacy. *Bifidobacterium breve (B*. *breve)* and *Bifidobacterium longum* (*B. longum*) significantly improve dendritic cell function and promote CD8^+^ T-cell activation, thereby enhancing responses to PD-1/PD-L1 blockade (Sivan et al., 2015). These findings suggest that microbiome-mediated enhancement of antigen presentation represents a central mechanism linking gut microbial composition to successful immunotherapy outcomes.

#### 3.2.2 Promotion of effector t-cell priming and th1-polarized antitumor immunity

A second major mechanism involves the induction of effector T-cell responses and Th1-polarized immunity. Species including *A. muciniphila*, *Alistipes indistinctus* (*A. indistinctus*), and *Enterococcus hirae* (*E. hirae*) have been associated with improved antigen-specific T-cell responses and enhanced responsiveness to PD-1 blockade (Griffin et al., 2021; Routy et al., 2018). Additional species such as *Enterococcus faecium* (*E. faecium*), *Enterococcus durans* (*E. durans*), *Enterococcus mundtii* (*E. mundtii*), *Coprobacillus cateniformis* (*C. cateniformis*), *Erysipelatoclostridium ramosum* (*E. ramosum*), *Lactobacillus gallinarum* (*L. gallinarum*), and *Lactobacillus rhamnosus* GG (*L. rhamnosus* GG) have similarly been shown to promote antitumor immune activation and improve PD-1/PD-L1-mediated tumor control (Fong et al., 2023; Griffin et al., 2021; Park et al., 2023).

Distinct microbial signatures have also been linked to enhanced responses to CTLA-4 blockade. Species such as *Bacteroides fragilis* (*B. fragilis*), *Bacteroides thetaiotaomicron* (*B. thetaiotaomicron*), *Burkholderia cepacia* (*B. cepacia*), *Bifidobacterium pseudolongum* (*B. pseudolongum*), *Lactobacillus johnsonii* (*L. johnsonii*), *Olsenella* spp., and *E. faecium* have been shown to promote antitumor immunity in anti-CTLA-4-treated mice (Griffin et al., 2021; Mager et al., 2020; Vétizou et al., 2015). These bacteria facilitate the development of immune environments characterized by increased IFN-γ production, enhanced T-cell infiltration, and improved tumor control.

#### 3.2.3 Microbial metabolite-mediated immune modulation

Beyond direct immune-cell activation, microbial metabolites also contribute to immunotherapy responsiveness. Butyrate-producing bacteria such as *Roseburia intestinalis* (*R. intestinalis*) and *F. prausnitzii* have been associated with favorable immunotherapy outcomes through modulation of immune cell differentiation, maintenance of epithelial barrier integrity, and regulation of inflammatory signaling pathways (Bredon et al., 2024; Kang et al., 2023). These metabolites influence both innate and adaptive immune responses and contribute to a tumor microenvironment that is more permissive to effective checkpoint blockade.

Clinical observations from selected patient cohorts further support this concept. Enrichment of Akkermansia has been associated with pathological complete response; however, these associations require validation across larger and more diverse clinical populations. Similarly, residual disease has been linked to increased abundance of tryptophan-metabolizing *Clostridium* species and elevated indole-derived metabolites. Another study showed that reduced abundance of butyrate-producing taxa including *Faecalibacterium* and *Roseburia*, increases the inflammatory mediators such as CXCL5 and CCL2. Which further support the existence of a microbiome–metabolite–immune axis governing immunotherapy response in triple-negative breast cancer (Sarfraz et al., 2024; Sarfraz et al., 2025).

#### 3.2.4 Antigen mimicry and microbiome-driven immune recognition

Emerging evidence suggests that certain gut microbes may enhance immune checkpoint efficacy through antigen mimicry and immune cross-reactivity. In this mechanism, microbial antigens share structural similarities with tumor-associated antigens, allowing microbe-primed T cells to recognize and attack tumor cells. Direct evidence for this phenomenon was provided by Fluckiger et al., who demonstrated cross-reactivity between an *E*. *hirae* bacteriophage-derived epitope and the tumor-associated antigen PSMB4, resulting in enhanced antitumor CD8^+^ T-cell responses (Fluckiger et al., 2020). Similarly, commensal bacteria have been shown to stimulate antitumor immunity through T-cell cross-reactivity against shared microbial and tumor antigens, further supporting a role for microbiome-driven immune recognition in cancer immunotherapy (Bessell et al., 2020). These findings support the concept that the microbiome contributes not only to immune activation but also to shaping immune recognition of tumors.

Importantly, these microbiome-mediated effects have been demonstrated across multiple experimental settings, including germ-free mice mono-colonized with specific bacterial strains (Mager et al., 2020; Park et al., 2023; Vétizou et al., 2015), antibiotic-treated mice subsequently recolonized via oral gavage (Routy et al., 2018; Vétizou et al., 2015), and conventionally housed mice receiving bacterial supplementation (Fong et al., 2023; Griffin et al., 2021; Kang et al., 2023; Sivan et al., 2015). Conversely, loss of therapeutic efficacy has been observed in germ-free animals, antibiotic-treated mice, and mice harboring distinct baseline microbiota depending on housing facility (Taconic Biosciences versus The Jackson Laboratory) (Mager et al., 2020; Park et al., 2023; Sivan et al., 2015; Vétizou et al., 2015). These observations provide compelling evidence that gut microbial composition critically shapes antitumor immune responses to ICIs.

Notably, microbiome-mediated immunomodulation remains highly context-dependent. Administration of certain probiotics, including *B. longum* 35,624 and *L. rhamnosus* GG, has been associated with impaired ICI responses in some settings (Spencer et al., 2021). Interestingly, *L. rhamnosus* GG has been reported to exert both beneficial and detrimental effects in different ICI models, suggesting that microbial function may depend on tumor type, checkpoint target, bacterial strain source, dosing strategy, and host microbial background. These findings underscore the complexity of host–microbe–therapy interactions and highlight the need for personalized microbiome-based interventions.

Collectively, these findings demonstrate that gut microbiota influences immune checkpoint efficacy through coordinated effects on antigen presentation, T-cell activation, microbial metabolite production, and immune recognition. Understanding these mechanistic pathways provides a framework for the rational development of microbiome-based strategies aimed at enhancing cancer immunotherapy. Importantly, microbiome composition and metabolite profiles may serve as predictive biomarkers of immune checkpoint inhibitor responsiveness and support patient stratification and personalized immunotherapy strategies. The major microbiome-mediated mechanisms influencing immune checkpoint blockade efficacy are summarized in Figure 2.

### 3.3 Microbial antigen mimicry and t-cell cross-reactivity

Microbial antigen mimicry refers to the phenomenon in which microbial antigens share structural or sequence similarity with tumor-associated antigens or neoantigens, resulting in T-cell cross-reactivity (Boesch et al., 2021). Emerging evidence suggests that microbiome-derived antigens can modulate antitumor immunity by priming T cells capable of recognizing both microbial and tumor epitopes (Ragone et al., 2022). In patients receiving immune checkpoint blockade (ICB) therapy, cross-reactivity between microbial xeno-antigens and tumor neoantigens has been shown to enhance antitumor immune responses and influence therapeutic efficacy. This mechanism has attracted considerable interest because it provides a direct link between microbial composition and tumor immune recognition. Recent studies have demonstrated that microbial antigen mimicry can promote antitumor immunity through expansion of cross-reactive CD8^+^ T-cell populations that recognize shared microbial and tumor antigens, thereby enhancing immune checkpoint blockade efficacy and tumor control (Bessell et al., 2020; Fluckiger et al., 2020).

*E. hirae* has been found to express a prophage peptide that triggers a major histocompatibility complex (MHC) class I immune response, which in turn cross-reacts with an epitope in glycerol-3-phosphate dehydrogenase 1-like protein (GPD1L), a protein overexpressed in tumor cells. This suggests that microbial peptides can alter tumor immune responses by modulating antigen presentation (Fluckiger et al., 2020). Studies have shown that certain commensal bacteria within the tumor microenvironment (TME) express human leukocyte antigen (HLA) class I and II-restricted peptides, allowing them to interact directly with tumor-infiltrating immune cells (Mohseni et al., 2023; Naghavian et al., 2023). In melanoma metastases, bacterial species have been identified within tumor cells, where they present peptides capable of eliciting T-cell recognition, indicating that intratumoral bacteria may directly shape tumor immunity (Du Toit, 2021).

Previous studies demonstrated that anti-CTLA-4 efficacy depends on gut commensals such as *B*. *fragilis* and *B*. *thetaiotaomicron*, and that adoptive transfer of *B. fragilis*-specific T cells can restore antitumor responses in microbiota-depleted mice (Vétizou et al., 2015). More direct evidence for molecular mimicry was subsequently provided by Fluckiger et al., who demonstrated cross-reactivity between an *E*. *hirae* bacteriophage epitope and the tumor-associated antigen PSMB4, resulting in enhanced antitumor CD8^+^ T-cell responses (Fluckiger et al., 2020). The discovery that bacterial peptides can mimic tumor antigens opens up new questions regarding the role of microbiomes in shaping anti-tumor immune responses. Some microbial peptides have been found to elicit protective immune responses, while others contribute to immune evasion by mimicking self-antigens and inducing immune tolerance (Riemer, 2021).

Microbial peptides have also been identified as contributors to immune-related toxicity in immune checkpoint blockade therapy. This occurs when microbial antigens activate tumor-reactive T cells, leading to autoimmune-like inflammation in treated patients (Naqash et al., 2021). The interplay between microbial antigen mimicry and tumor immune evasion presents challenges and opportunities for cancer immunotherapy. While some microbial peptides enhance immune responses, others contribute to immune suppression, complicating the use of immune checkpoint inhibitors (ICIs).

Additionally, identifying microbial-derived tumor-associated antigens could open new avenues for microbiome-based cancer vaccines and precision immunotherapy (Zhang et al., 2024). Understanding how bacteria within the TME interact with the immune system is critical for developing effective microbiome-targeted interventions, which may include probiotic therapies, engineered bacterial vaccines, and microbial metabolite-based drugs aimed at enhancing tumor immune surveillance (Akbariqomi et al., 2025). The microbiome is a powerful regulator of immune responses and plays a fundamental role in tumor immunology. By promoting chronic inflammation, influencing immune checkpoint pathways, and mimicking tumor antigens, microbes shape cancer progression and therapeutic responses. Understanding the complex interactions between microbiota and the immune system offers potential opportunities for developing microbiome-informed cancer immunotherapies. However, most microbiome-targeted approaches, including probiotics, dietary interventions, and fecal microbiota transplantation, remain at preclinical or early clinical stages and require rigorous validation before routine clinical implementation.

## 4 Microbiome and tumor microenvironment (TME)

The tumor microenvironment (TME) is a dynamic and heterogeneous milieu consisting of cancer cells, immune cells, stromal components, and microbial communities that influence tumor growth, immune evasion, and response to therapy. Increasing evidence suggests that tumor-associated microbiota play a pivotal role in shaping immune suppression, modulating metabolic signaling, and enhancing tumor progression (Li et al., 2025). Microbial colonization of tumors and their secreted metabolites contribute significantly to the immune and metabolic landscape of the TME, thereby affecting therapeutic efficacy. This section explores mechanisms by which microbiota directly colonize tumors and how their metabolites influence immune regulation and tumorigenesis.

### 4.1 Direct microbial colonization of tumors: tumor-associated microbiota modulating local immune suppression

Recent advances in sequencing and imaging technologies have revealed that bacteria can reside within tumor tissues, forming a distinct tumor-associated microbiota that directly interacts with cancer cells and immune populations in the tumor microenvironment. Unlike systemic effects mediated by the gut microbiome, intratumoral microbes exert local influences on tumor biology by modulating immune signaling, inflammatory pathways, and cellular stress responses, thereby contributing to tumor progression and therapeutic resistance. Comprehensive analyses of intratumoral microbial communities have demonstrated that bacteria within tumors can alter local immune surveillance by impairing antigen presentation, skewing myeloid cell recruitment, and suppressing cytotoxic T-cell function. These effects collectively promote immune tolerance and enable tumor persistence.

Recent reviews and experimental studies highlight that tumor-associated microbes can shape the immune landscape through direct interactions with dendritic cells, macrophages, and T lymphocytes, influencing both innate and adaptive immune responses within tumors (Tingting et al., 2025). Among tumor-enriched bacteria, *F. nucleatum* has emerged as one of the most extensively characterized examples of direct microbial involvement in tumor progression, particularly in colorectal cancer (CRC). Multiple studies have reported a preferential enrichment of *F. nucleatum* within CRC tissues, where its presence correlates with poor prognosis, increased metastatic potential, and resistance to chemotherapy. Mechanistically, *F. nucleatum* promotes tumor progression through direct interactions with tumor cells. Rubinstein and colleagues demonstrated that the bacterial adhesin FadA binds to E-cadherin on colorectal epithelial cells, leading to activation of β-catenin signaling and induction of oncogenic and inflammatory gene expression programs that facilitate tumor cell proliferation and invasion (Rubinstein et al., 2013).

These findings establish a direct molecular link between intratumoral bacteria and tumor-intrinsic signaling pathways. In addition to tumor cell-autonomous effects, *F. nucleatum* reshapes the local immune microenvironment. Kostic et al. showed that fusobacterial colonization in intestinal tumor models is associated with increased recruitment of tumor-infiltrating myeloid cells and establishment of a pro-inflammatory yet immunosuppressive milieu that supports tumor growth (Kostic et al., 2013). This altered immune contexture limits effective antitumor immune responses despite the presence of inflammatory signaling. Importantly, intratumoral *F. nucleatum* has also been implicated in chemotherapy resistance. Yu et al. demonstrated that *F. nucleatum* activates Toll-like receptor 4 (TLR4) and MYD88 signaling in colorectal cancer cells, leading to the regulation of specific microRNAs that promote autophagy and protect tumor cells from chemotherapy-induced apoptosis (Yu et al., 2017). This autophagy-mediated survival mechanism provides a direct link between tumor-associated bacteria and therapeutic failure.

*F*. *nucleatum*, a bacterial species frequently found in colorectal cancer (CRC), has been shown to interact with immune inhibitory receptors such as T-cell immunoreceptor with immunoglobulin and immunoreceptor tyrosine-based inhibitory motif domains (TIGIT) and CEACAM1, which suppress NK cell and T-cell cytotoxic responses. This interaction dampens anti-tumor immune activity and promotes tumor progression (Gur et al., 2015; 2019; Kim et al., 2019; Zhang et al., 2018).

While bacterial species represent the most extensively studied members of the tumor-associated microbiota, emerging evidence suggests that fungal communities (the tumor mycobiome) can also contribute to tumor progression through local immune modulation and inflammatory signaling. In pancreatic cancer, *Malassezia* species have been found within the TME, where they activate complement pathways via mannose-binding lectin interactions, promoting tumor progression (Brayer et al., 2023; Zhang et al., 2025). Additionally, *Candida* species found in gastrointestinal tumors have been linked to worse survival outcomes and increased metastatic potential, suggesting that tumor-associated fungal communities contribute to immune suppression, chronic inflammation, and metastatic progression within the tumor microenvironment (Zhang et al., 2022). Collectively, these studies support a model in which direct microbial colonization of tumors contributes to cancer progression by simultaneously activating oncogenic signaling pathways, remodeling the local immune microenvironment toward immune suppression, and inducing resistance to anticancer therapies. Targeting tumor-associated microbiota may represent a potential therapeutic avenue; however, clinical translation remains limited and requires further mechanistic and clinical validation.

### 4.2 Microbial metabolites shaping the tumor microenvironment: scfa-mediated immunometabolic regulation

SCFAs particularly butyrate (C4) and propionate (C3) are dominant microbial fermentation products of dietary fiber in the colon and are now recognized as potent immunometabolic signals that tune the balance between inflammation and tolerance. A central outcome of SCFA signaling is the expansion and functional reinforcement of Foxp3^+^ regulatory T cells (Tregs), a cell type that strongly influences the tumor microenvironment (TME) by controlling inflammation but also, in many cancers, by suppressing anti-tumor immunity.

Three landmark studies established causality between SCFAs and Treg biology: Furusawa et al. showed butyrate enhances Foxp3 locus acetylation and drives colonic Treg differentiation (Furusawa et al., 2013). Arpaia et al. demonstrated that both butyrate and propionate promote peripheral (extrathymic) Treg generation in a CNS1-dependent manner (Arpaia et al., 2013), and Smith et al. showed SCFAs regulate the size/function of the colonic Treg pool via FFAR2/GPR43 signaling *in vivo* (Arpaia et al., 2013; Furusawa et al., 2013; Smith et al., 2013).

#### 4.2.1 Mechanistic basis: epigenetic licensing and receptor-mediated signaling

##### 4.2.1.1 HDAC inhibition and chromatin remodeling at the Foxp3 program

Butyrate is a well-characterized histone deacetylase (HDAC) inhibitor, and direct epigenetic remodeling is a key mechanism by which it promotes the Treg lineage. In Furusawa et al., butyrate enhanced histone H3 acetylation at the promoter and conserved non-coding sequence (CNS) regions of the Foxp3 locus under Treg-polarizing conditions, supporting stable induction of Foxp3 expression (Furusawa et al., 2013).

Arpaia et al. further showed that butyrate increases Foxp3 protein acetylation, which stabilizes Foxp3 and can enhance suppressive function; importantly, they observed that propionate can also potentiate peripheral Treg generation, consistent with a shared HDAC-inhibitory axis (Furusawa et al., 2013).

##### 4.2.1.2 SCFA sensing through GPCRs (FFAR2/GPR43; GPR109A) and immune conditioning

*In vivo*, SCFA effects are also mediated through cell-surface receptors expressed on immune and epithelial compartments. Smith et al. demonstrated that SCFAs regulate colonic Tregs in a manner dependent on FFAR2 (GPR43), linking microbial metabolites to adaptive immune calibration (Smith et al., 2013).

Butyrate is also a ligand for GPR109A (NIACR1). Work identifying this axis in colon biology suggests a pathway by which butyrate can foster a more tolerogenic environment partly by shaping antigen-presenting cells (APCs) to support Treg and IL-10 programs and thereby influence inflammation-associated tumorigenesis (Singh et al., 2014; Thangaraju et al., 2009).

##### 4.2.1.3 Context dependence

SCFAs can promote regulatory *and* effector programs. A recurring theme is that SCFAs do not uniformly “turn on tolerance”; they bias T-cell differentiation depending on the cytokine milieu and activation context. Park et al. showed that SCFAs can promote differentiation into effector lineages (e.g., Th1/Th17) alongside IL-10–associated regulatory states via HDAC inhibition and mTOR–S6K pathway modulation (Park et al., 2015). This context dependence is crucial when extrapolating SCFA-Treg biology into tumors (Park et al., 2015).

#### 4.2.2 Implications for the tumor microenvironment

Tregs are often enriched in tumors and correlate with immune suppression, reduced effector T-cell activity, and therapy resistance in multiple settings. Within gastrointestinal cancers, especially colorectal cancer (CRC)—SCFAs are uniquely positioned to influence the TME because luminal concentrations are high and epithelial/immune compartments are continuously exposed. In inflammation-driven carcinogenesis models, SCFA receptor pathways appear protective: for example, FFAR2/GPR43 signaling is linked to suppression of intestinal carcinogenesis and is reported to be down-modulated in human colon cancers (Sivaprakasam et al., 2016).

However, from an immuno-oncology perspective, the same SCFA-driven increase in Treg stability and function could also raise the “regulatory tone” of the TME, potentially dampening anti-tumor cytotoxic responses particularly if the tumor is already Treg-dominant and metabolically permissive to suppressive programs.

A useful synthesis is that butyrate/propionate-driven Treg induction can be beneficial or detrimental depending on tumor context:

- Beneficial (anti-tumor, indirectly): by suppressing chronic inflammation that fuels tumor initiation (notably in colitis-associated CRC), and by maintaining epithelial integrity and immune homeostasis through receptor-linked pathways (e.g., GPR109A/FFAR2) (Singh et al., 2014).
- Potentially detrimental (pro-tumor, in established tumors): by strengthening Treg stability (via Foxp3 acetylation and epigenetic reinforcement) and sustaining immunosuppressive cytokine networks, which may restrain effector priming and intratumoral killing (Arpaia et al., 2013).

#### 4.2.3 Translational considerations

These mechanistic insights suggest several intervention angles dietary fiber modulation, SCFA delivery (or pro-drugs), and targeting SCFA receptors but they also argue for precision use: increasing SCFAs might help prevent inflammation-associated tumorigenesis, while in patients with established, immune-cold tumors, indiscriminate enhancement of Treg programs could be counterproductive. A practical recommendation for future studies is to pair SCFA measurements (stool/serum), microbiome functional capacity, and tumor immune profiling (Foxp3^+^ Tregs, CD8^+^ infiltration, APC activation states) to determine when butyrate/propionate act predominantly as homeostatic protectors versus immunosuppressive amplifiers (Arpaia et al., 2013; Furusawa et al., 2013; Smith et al., 2013).

These findings suggest that SCFA levels, microbial functional capacity, and tumor immune profiles may collectively serve as biomarkers for patient stratification. Such approaches may help identify individuals most likely to benefit from dietary interventions, microbiome modulation, or immunotherapeutic strategies targeting the tumor immune microenvironment.

### 4.3 Microbial toxins inducing dna damage and tumor progression

A subset of bacterial virulence factors has been experimentally shown to contribute to carcinogenesis by directly inducing DNA damage or by activating host inflammatory programs that promote epithelial hyperplasia and tumor formation. Among the most well-supported examples are:

- Colibactin, produced by *pks* ^+^ *E. coli*, which is associated with DNA damage and a distinct mutational signature in human colorectal cancer genomes;
- *B. fragilis* toxin (BFT) from enterotoxigenic *B. fragilis* (ETBF), which promotes tumorigenesis through STAT3/Th17-dependent inflammatory pathways and disrupts epithelial junctional integrity *via* E-cadherin cleavage; and
- CagA from *H. pylori*, which is translocated into host cells through a type IV secretion system and has direct *in vivo* oncogenic evidence from transgenic mouse models.

#### 4.3.1 Colibactin and colorectal cancer

Colibactin is produced by *E. coli* strains carrying the pks pathogenicity island. Experimental models demonstrate that exposure to *pks* ^+^ bacteria induce DNA damage in intestinal epithelial cells, and that prolonged exposure can result in persistent genomic alterations. In a colitis-associated colorectal cancer (CRC) model, Arthur *et al.* reported that intestinal inflammation modulates microbiota-driven tumorigenic activity and identified a cancer-promoting contribution from colibactin-producing bacteria within this inflammatory context (Arthur et al., 2012).

A direct link between colibactin exposure and somatic mutagenesis was established by Pleguezuelos-Manzano et al., who repeatedly exposed human intestinal organoids to genotoxic *pks* ^+^ *E. coli* via luminal injection over a five-month period, followed by whole-genome sequencing of clonal organoids. They reported that this exposure produced a distinct mutational signature that was absent in organoids exposed to isogenic *pks*-mutant bacteria. Importantly, the same mutational signature was identified in a subset of 5,876 human cancer genomes, with predominant enrichment in colorectal cancer. The study further describes that *pks* encodes enzymes responsible for colibactin biosynthesis and discusses DNA alkylation particularly on adenine residues as a plausible mechanism underlying the observed DNA damage markers in exposed epithelial cells (Pleguezuelos-Manzano et al., 2020). Together, these studies provide experimental and genomic evidence linking colibactin-producing *E. coli* to DNA damage and mutational processes relevant to colorectal carcinogenesis.

#### 4.3.2 *B*. *fragilis* toxin (BFT) and tumor invasion

*B. fragilis* has been implicated in colorectal carcinogenesis primarily through the activity of *B. fragilis* toxin (BFT), which is secreted by enterotoxigenic *B. fragilis* (ETBF). Primary studies support two interconnected observations: (i) ETBF colonization promotes tumor formation through STAT3-and Th17-dependent inflammatory pathways, and (ii) BFT directly disrupts epithelial junctional architecture via E-cadherin cleavage. In a landmark *in vivo* study, Wu et al. demonstrated that although both ETBF and nontoxigenic *B. fragilis* strains chronically colonized mice, only ETBF induced colitis and robust colonic tumor formation in *Min* mice. The authors further reported selective activation of colonic STAT3 signaling and a dominant Th17 immune response in ETBF-colonized animals. Antibody-mediated blockade of IL-17 or the IL-23 receptor significantly reduced ETBF-induced colitis, epithelial hyperplasia, and tumor formation, supporting a STAT3/Th17-dependent mechanism of inflammation-driven tumorigenesis (Wu et al., 2009).

At the epithelial level, Wu *et al.* demonstrated that BFT specifically cleaves the extracellular domain of E-cadherin, with cleavage detectable within 1 minute of toxin exposure. The authors report that this cleavage is essential for the morphological and physiological effects of BFT observed in their experimental system (Wu et al., 1998). Collectively, these findings support a model in which ETBF-associated tumorigenesis involves both host inflammatory circuitry required for tumor development *in vivo* and a toxin-mediated disruption of epithelial adhesion.

#### 4.3.3 *H*. *pylori* CagA and gastric cancer

Chronic infection with *H. pylori* is a well-established risk factor for gastric cancer, particularly in strains expressing cytotoxin-associated gene A (CagA). CagA is delivered into gastric epithelial cells through a type IV secretion system, where it alters host signaling pathways involved in cell proliferation and survival.

A detailed mechanistic synthesis by Hatakeyama (2017) summarizes evidence that CagA undergoes tyrosine phosphorylation at EPIYA motifs following translocation into host cells and functions as a non-physiological signaling scaffold by interacting with host proteins, including SHP2 and PAR1/MARK. The review further notes that transgenic expression of CagA in experimental models confirms its oncogenic potential (Hatakeyama, 2017; Peinado et al., 2017).

Primary *in vivo* evidence was provided by Ohnishi *et al.*, who generated transgenic mice expressing either wild-type or phosphorylation-resistant CagA. The authors reported that mice expressing wild-type CagA developed gastric epithelial hyperplasia, with a subset progressing to gastric polyps and adenocarcinomas of the stomach and small intestine. Additional systemic effects, including hematologic abnormalities and malignancies, were observed. Importantly, these pathological outcomes were not observed in mice expressing phosphorylation-resistant CagA, highlighting the requirement for CagA tyrosine phosphorylation (Ohnishi et al., 2008).

#### 4.3.4 Other microbial toxins and emerging evidence

Beyond colibactin, BFT, and CagA, emerging evidence implicates additional bacterial pathogens in carcinogenesis. Scanu *et al.* investigated the association between chronic *S. Typhi* infection and gallbladder carcinoma by experimentally testing causality and mechanism. They reported that *S. enterica* induced malignant transformation in genetically predisposed mice, murine gallbladder organoids, and fibroblasts, accompanied by TP53 mutations and c-MYC amplification. The study further identified MAPK and AKT pathway activation, mediated by bacterial effectors secreted during infection, as critical for initiating and sustaining transformation, leading the authors to conclude that *Salmonella* can act as a causative agent in gallbladder carcinoma within their experimental framework (Scanu et al., 2015).

While the studies summarized above differ in experimental models and tissue context, they collectively highlight microbial toxins as active drivers rather than passive correlations of tumorigenesis. Unlike generalized dysbiosis, these toxins exhibit defined molecular activities such as DNA alkylation, junctional protein cleavage, or intracellular signal rewiring that directly intersect with canonical cancer pathways. A recurring theme across colibactin, *B.* I toxin (BFT), and CagA is the convergence of genomic instability and inflammatory signaling, suggesting that microbial toxins may act at the interface of mutation accumulation and tumor-promoting microenvironments. Additional bacterial toxins and virulence factors with emerging or context-dependent carcinogenic evidence are summarized in Table 1, further supporting the concept that microbe-derived genotoxic and signaling activities contribute to cancer development across diverse biological settings.

**TABLE 1:** Bacterial toxin and virulence factors involved in carcinogenesis.

Bacterial factor	Bacterial origin	Primary mechanism (as reported)	*In vivo* evidence	Key references (DOI)
Colibactin	*E. coli* (pks^+^ strains)	Induces DNA double-strand breaks; forms DNA adducts and interstrand cross-links; associated with a distinct mutational signature in host genomes	Yes – promotes tumors in mouse models; mutational signature detected in human CRC genomes	Arthur et al. (2012), Pleguezuelos-Manzano et al. (2020)
*Bacteroides fragilis* toxin (BFT/fragilysin)	Enterotoxigenic*B*. *fragilis* (ETBF)	Zinc-dependent metalloprotease; cleaves extracellular E-cadherin; activates STAT3 and Th17-driven inflammation	Yes – induces colitis, epithelial hyperplasia, and colon tumors in *Min* mice	Wu et al. (1998), Wu et al. (2009)
CagA	*H. pylori* (cagA^+^ strains)	Injected via type IV secretion system; undergoes tyrosine phosphorylation; interacts with SHP2 and PAR1/MARK; perturbs epithelial polarity and signaling	Yes – gastric hyperplasia and adenocarcinoma in CagA-transgenic mice	Ohnishi et al. (2008)
Cytolethal distending toxin (CDT)	*Campylobacter*, *H. hepaticus*, some *E. coli*	DNase-like activity (CdtB subunit); induces DNA double-strand breaks and chromosomal instability	Yes - liver and intestinal tumors in chronic infection mouse models	Ge et al. (2007)
Salmonella effector proteins (T3SS-delivered)	*S. enterica serovar Typhi*	Activates MAPK and AKT signaling; induces genomic instability in predisposed cells	Yes - malignant transformation in mice and gallbladder organoids	Scanu et al. (2015)
Cytotoxic necrotizing factor 1 (CNF1)	Uropathogenic *E. coli* (UPEC)	Deamidates Rho GTPases; induces cytoskeletal remodeling, proliferation, and migration	No direct tumor formation; pro-tumorigenic phenotypes in cellular and infection models	Guo et al. (2020)
FadA adhesin	*F*. *nucleatum*	Binds E-cadherin; activates β-catenin signaling and epithelial proliferation	Yes - tumor promotion in CRC mouse models	Gur et al. (2013)
Fap2 (immune inhibitory protein)	*F. nucleatum*	Binds TIGIT on immune cells; suppresses anti-tumor immunity	Yes - promotes immune evasion in CRC models	Gur et al. (2015)

Importantly, the strength of evidence linking microorganisms to cancer varies substantially across microbial taxa and should be interpreted accordingly. Among the examples discussed, *H. pylori* represent one of the strongest cases for a causal role in human carcinogenesis, supported by extensive epidemiological evidence, mechanistic studies, and direct oncogenic effects demonstrated in animal models. In contrast, organisms such as *F*. *nucleatum* and colibactin-producing *E*. *coli* are supported by compelling mechanistic and translational evidence implicating them in tumor progression, immune modulation, and mutagenesis, although their roles as primary carcinogenic drivers remain less firmly established. For many additional microbial taxa identified through sequencing-based studies, current evidence remains largely associated. Therefore, distinguishing causal, mechanistic, and correlative relationships remains a central challenge in microbiome-oncology research and highlights the need for longitudinal studies, functional validation, and experimental model systems to establish biological causality. Beyond their mechanistic roles in tumorigenesis, microbial toxins and virulence factors may also have important clinical applications as biomarkers of microbiome-driven cancer risk, disease progression, and therapeutic responsiveness. Integration of microbial virulence profiles, toxin-associated mutational signatures, and host genomic information may facilitate patient stratification and support the development of precision microbiome-targeted interventions. Future studies integrating microbial genomics, host mutational profiling, and spatial tumor ecology will be essential to determine when toxin-producing microbes initiate tumorigenesis versus accelerate established disease. A summary of major bacterial toxins and their oncogenic mechanisms is provided in Table 1.

## 5 Microbiome-mediated drug metabolism

The microbiome plays a crucial role in modulating drug metabolism, influencing the efficacy and toxicity of various cancer therapies. The microbiome can shape cancer therapy outcomes through two experimentally supported routes: direct biotransformation of drugs (or drug conjugates) by microbial enzymes, and indirect control of therapy efficacy/toxicity via microbiome-dependent immune and inflammatory programs. In the direct route, microbial enzymes can reactivate detoxified drug metabolites or inactivate active agents, shifting local exposure and toxicity or generating resistance. In the indirect route, microbiota perturbations (e.g., antibiotics, dysbiosis) alter cytokine tone, antigen presentation, myeloid cell function, and T cell priming, thereby changing the antitumor effect of chemotherapy and immunotherapy, and modulating tissue injury responses after radiation (Geller et al., 2017; Iida et al., 2013; Viaud et al., 2013; Wallace et al., 2010).

### 5.1 Microbiome-mediated drug metabolism leading to toxicity or therapeutic failure

One of the most well-characterized examples of microbiome-mediated toxicity involves irinotecan (CPT-11). Wallace et al. demonstrated that gut bacterial β-glucuronidases hydrolyze the hepatic detoxification product SN-38G back into the active metabolite SN-38 in the intestinal lumen, causing epithelial damage and dose-limiting diarrhea. Importantly, the authors showed that selective inhibition of bacterial β-glucuronidases reduced gastrointestinal toxicity in mice without broadly disrupting the microbiota or compromising anticancer efficacy (Wallace et al., 2010).

Direct microbial inactivation of chemotherapeutic agents has also been demonstrated. Geller et al. reported that intratumoral *Gammaproteobacteria* express a long isoform of cytidine deaminase that metabolizes gemcitabine into its inactive form, conferring resistance in a mouse colon cancer model. Antibiotic treatment restored gemcitabine sensitivity, directly implicating bacterial drug metabolism in therapeutic failure (Geller et al., 2017). Extending this concept, Lehouritis et al. systematically examined interactions between bacteria and multiple chemotherapeutic drugs and showed that local bacteria can chemically modify or degrade anticancer agents, thereby reducing or, in some cases, enhancing cytotoxic efficacy *in vitro* and in murine tumor models.

These findings indicate that microbial drug metabolism is not limited to a single compound but may broadly influence chemotherapy responses (Lehouritis et al., 2015). More recently, Spanogiannopoulos et al. showed that a conserved bacterial preTA operon enables gut bacteria to metabolize 5-fluorouracil (5-FU) to dihydrofluorouracil (DHFU), and they report that this activity can reduce fluoropyrimidine efficacy in mouse experiments and that preTA is prevalent in gut metagenomes from colorectal cancer cohorts (Spanogiannopoulos et al., 2022). Radiotherapy can also induce microbiome-associated adverse effects. Gerassy-Vainberg et al. report that rectal irradiation alters luminal and mucosa-associated microbiota, and that transferring post-irradiation microbiota to germ-free mice increases susceptibility to radiation injury and DSS colitis, supporting microbiota-mediated transmission of inflammatory susceptibility (Gerassy-Vainberg et al., 2018). Study highlighting microbiome mediated modulation of chemotherapeutics agents are listed in Table 2.

**TABLE 2:** Microbiome-mediated modulation of cancer therapy through drug metabolism and immune regulation.

Therapy	Microbial factor	Key microbes (as stated in paper)	Mechanism (as stated)	Clinical impact	References
Irinotecan	Bacterial β-glucuronidase	Gut bacterial GUS (multiple taxa)	Deconjugation/reactivation of SN-38G contributing to GI toxicity	Diarrhea/GI toxicity	Wallace et al. (2010)
Irinotecan	β-glucuronidase inhibitor	Gut bacterial GUS	GUS activity linked to irinotecan metabolism/toxicity; inhibitor work reported	Improved tolerability (preclinical)	Lin et al. (2021)
5-FU	Drug inactivation (preTA)	*Proteobacteria* and *Firmicutes* (broad); *E. coli* preTA shown	5-FU → DHFU *via* preTA; reduces bioavailability/efficacy in mice; prevalent in CRC gut microbiomes	Reduced fluoropyrimidine efficacy	Spanogiannopoulos et al. (2022)
Cyclophosphamide	Microbiota dependence + translocation	Gram + species (selected)	CTX alters microbiota; induces translocation of selected Gram + to secondary lymphoid organs	Supports antitumor immune efficacy (mouse models)	Viaud et al. (2013)
Cyclophosphamide	Species-level “oncomicrobiotics”	*E. hirae*, *B. intestinihominis*	*E. hirae* translocation; *B. intestinihominis* promotes intratumoral immune features; memory responses predict PFS in patient cohort (as reported)	Enhanced efficacy/biomarker association	Daillère et al. (2016)
Radiotherapy (rectal)	Dysbiosis + transmissible susceptibility	Not restricted to 1–2 taxa in the paper’s main conclusion	Rectal radiation induces dysbiosis; irradiated microbiota transmits radiation/inflammatory susceptibility; IL-1β implicated	GI injury/inflammation susceptibility	Gerassy-Vainberg et al. (2018)
Anti-PD-1	Microbiome association	Diverse taxa (cohort-level)	Responders vs. non-responders differ in diversity/composition	Better response	Gopalakrishnan et al. (2018)
Anti-CTLA-4	Th1 priming/*Bacteroides* dependence	*Bacteroides* spp. incl. *B. fragilis*	CTLA-4 blockade efficacy depends on gut microbiota; *Bacteroides*-specific responses reported	Enhanced efficacy	Vétizou et al. (2015)
ICB (anti-PD-1)	FMT + reinduction	Responder-derived microbiota	Clinical trial: FMT + anti-PD-1 changed gut microbiome and TME; responses in subset	Restored response in some non-responders	Davar et al. (2021)

### 5.2 Microbiome-mediated enhancement of drug efficacy and therapeutic benefit

In contrast to these detrimental effects, multiple studies demonstrate that microbiomes can enhance anticancer efficacy, particularly through immune-mediated mechanisms. Recent study by Viaud et al. showed that cyclophosphamide alters gut microbial composition and increases intestinal permeability, promoting the translocation of selected Gram-positive bacteria to secondary lymphoid organs. This bacterial translocation was associated with the induction of helper T cell responses required for optimal antitumor immunity in mouse models, establishing a microbiome-dependent component of cyclophosphamide efficacy (Viaud et al., 2013). Similarly, Iida et al. demonstrated that disruption of the gut microbiota impaired the antitumor effects of platinum-based chemotherapy (oxaliplatin and cisplatin) in mice. The study linked microbiome integrity to therapy-induced activation of tumor-infiltrating myeloid cells and reactive oxygen species production, indicating that commensal bacteria are necessary for full chemotherapeutic efficacy (Iida et al., 2013).

The strongest clinical and mechanistic evidence for beneficial microbiome involvement comes from immune checkpoint blockade (ICB). Routy et al. reported that antibiotic exposure was associated with reduced clinical benefit from PD-1/PD-L1 blockade in cancer patients and demonstrated that fecal microbiota transplantation (FMT) from responding patients restored antitumor responses in mouse models (Routy et al., 2018). Independent melanoma cohorts confirmed these observations. Gopalakrishnan et al. showed that higher gut microbiome diversity and specific bacterial community structures were associated with improved responses to anti-PD-1 therapy, with functional validation in germ-free mice receiving responder-derived microbiota (Gopalakrishnan et al., 2018).

Crucially, early interventional clinical trials provide proof-of-concept that microbiome manipulation can overcome therapeutic resistance. Baruch et al. and Davar et al. independently reported that FMT combined with anti-PD-1 therapy induced clinical responses in a subset of patients with PD-1-refractory metastatic melanoma, establishing microbiome modulation as a viable therapeutic strategy (Baruch et al., 2021; Davar et al., 2021).

## 6 Microbiome as a diagnostic and prognostic biomarker

Recent advances in microbiome profiling have established the human microbiome as a potential source of diagnostic and prognostic biomarkers in cancer. High-throughput sequencing technologies, including 16S rRNA gene sequencing and whole-metagenome shotgun sequencing, have enabled systematic comparisons of microbial communities between cancer patients and non-cancer controls across multiple body sites. These studies demonstrate that specific microbial taxa and community patterns are reproducibly associated with particular cancer types, supporting their use as non-invasive biomarkers. In parallel, the detection of circulating microbial DNA in blood has emerged as a complementary liquid biopsy approach, while machine-learning frameworks have been applied to microbiome datasets to improve classification accuracy and risk prediction. This section summarizes evidence for microbiome-based cancer diagnostics across fecal, salivary, and blood-derived samples, and highlights computational approaches that leverage microbial signatures for cancer detection. Studies revealing diagnostic use of microbiomes are listed in Table 3.

**TABLE 3:** Microbial signature as diagnostic tools for detection of different cancer types.

Sample type	Cancer type	Key microbial signatures	Direction of change	Analytical approach	Clinical utility	Key references (DOI)
Feces	Colorectal cancer (CRC)	*F. nucleatum*, *B*. *fragilis*, *P*. *anaerobius*	Increased	Shotgun metagenomics, microbial gene markers	Early CRC detection; complements colonoscopy and FOBT	Shen et al. (2025), Wong et al. (2017)
Feces	Colorectal cancer (CRC)	*F*. *prausnitzii*	Decreased	Metagenomic sequencing	Loss of butyrate producers linked to carcinogenesis	Bautista et al. (2026)
Feces	Colorectal cancer (CRC)	*R*. *bicirculans* and *F*. *prausnitzi*	Discriminatory pattern	Machine learning on metagenomes	High sensitivity and specificity for CRC screening	Piccinno et al. (2025)
Saliva	OSCC/HNSCC	*P*. *gingivalis*, *F*. *nucleatum*	Increased	16S rRNA/metagenomics	Non-invasive oral cancer screening	Wu et al. (2025)
Saliva	Pancreatic cancer	*P*.*gingivalis, E nodatum, and P*. *micra*	Increased	Salivary microbiome profiling	Early detection and risk stratification	Meng et al. (2025)
Saliva	Pancreatic cancer	*Leptotrichia to Porphyromonas ratio*	Increased	Salivary microbial profiling	Differentiation from healthy controls	Oldford and Marshall (2015)
Blood (microbial EVs)	Pan-cancer	*Ruminococcaceae UCG-014*, *Lachnospiraceae NK4A136 group*, *Akkermansia*, *Turicibacter*, *Ruminiclostridium*, and *Lachnospiraceae UCG-001*	Increased	16s rRNA sequencing	Non-invasive liquid biopsy	Kim et al. (2021)
Blood (plasma)	Lung cancer	*Microvirgula aerodenitrificans (M. aerodenitrificans), K*.*phaffii, Alternaria incomplexa, Ogataea philodendra and S*.*s aureus*	Increased	Blood microbiome profiling	Disease detection and monitoring	Chen et al. (2025)
Feces + Host data	Colorectal cancer (CRC)	Multi-taxa microbial features	Predictive pattern	Random forest, SVM	Improved CRC classification	Gao et al. (2022)
Microbiome + Transcriptome	Pancreatic, lung cancer	Integrated microbial + host signatures	Enhanced prediction	Deep learning models	Prognosis and Therapeutics	Zhang et al. (2024)

### 6.1 Microbial signatures for early cancer detection

#### 6.1.1 Fecal microbiome analysis for colorectal cancer screening

Colorectal cancer (CRC) is among the most extensively studied malignancies concerning microbiome alterations, with distinct microbial signatures in fecal samples serving as potential biomarkers for early detection. The gut microbiota undergoes significant compositional shifts during colorectal carcinogenesis, with an enrichment of certain pathogenic bacteria and a depletion of commensal species. Studies have consistently demonstrated an increased abundance of *Fusobacterium nucleatum (F*. *nucleatum)*, *B. fragilis*, and *P*. *anaerobius* in CRC patients, while beneficial butyrate-producing bacteria such as *F. prausnitzii* are significantly reduced (Marcel et al., 2013). These microbial alterations contribute to colorectal carcinogenesis through mechanisms involving chronic inflammation, DNA damage, and immune evasion. The use of fecal microbiome analysis as a diagnostic tool has demonstrated high sensitivity and specificity in CRC detection, making it a valuable alternative or complement to traditional screening methods such as colonoscopy and fecal occult blood tests. Studies employing metagenomic sequencing have identified microbial gene markers capable of differentiating CRC patients from healthy individuals with high accuracy (Bettegowda et al., 2014). These findings suggest that microbiome-based fecal testing could significantly improve early detection strategies, potentially identifying high-risk individuals before tumor development.

#### 6.1.2 Salivary microbiome in oral and pancreatic cancer diagnostics

Beyond the gut, the oral microbiome has gained increasing recognition as a diagnostic tool for various malignancies, including oral, head and neck, and pancreatic cancers. The salivary microbiome is characterized by a diverse community of bacteria, with shifts in microbial composition often reflecting underlying pathological conditions. In oral squamous cell carcinoma (OSCC) and head and neck squamous cell carcinoma (HNSCC), an enrichment of *P. gingivalis* and *F. nucleatum* has been observed, indicating a potential role for these bacteria in tumor initiation and progression (Goto et al., 2016). In pancreatic cancer, salivary microbiome analysis has revealed distinct microbial profiles that differentiate cancer patients from healthy controls. Notably, a reduction in *Neisseria elongata* and *S. mitis*, coupled with an increase in *Porphyromonas* and *Granulicatella*, has been reported in pancreatic cancer patients. These microbial shifts suggest that salivary microbiome profiling could serve as a non-invasive screening method for pancreatic malignancies, potentially aiding in early detection and risk stratification.

### 6.2 Circulating microbial DNA as a liquid biopsy tool

#### 6.2.1 Blood microbiome profiling for non-invasive cancer diagnostics

Recent advancements in microbiome research have highlighted the potential of circulating microbial DNA (cmDNA) as a liquid biopsy tool for cancer detection. The presence of microbial DNA fragments in blood plasma provides a unique opportunity to explore systemic microbial alterations associated with malignancies. Studies have demonstrated that distinct microbial signatures can be detected in the blood of cancer patients, offering a non-invasive approach for early diagnosis and monitoring disease progression.

In lung cancer, an altered blood microbiome profile characterized by an increased prevalence of *Veillonella* and *Streptococcus* species has been observed (Kwiatkowska et al., 2025). Furthermore, machine learning models trained on microbial DNA sequencing data have successfully identified pan-cancer microbial signatures, demonstrating the potential of cmDNA-based diagnostics in multiple cancer types. The ability to detect tumor-associated microbial DNA in circulation provides a promising avenue for non-invasive cancer diagnostics, reducing the need for invasive tissue biopsies and enabling real-time monitoring of treatment responses.

### 6.3 Machine learning in microbiome-based cancer detection

#### 6.3.1 Predictive models integrating microbiomes and host transcriptomic data

The integration of machine learning with microbiome sequencing and host transcriptomic data has significantly advanced cancer detection accuracy. Computational models have been developed to analyze complex microbial patterns, correlating them with cancer risk, progression, and response to treatment. Supervised learning algorithms, including random forests, support vector machines (SVM), and neural networks, have been applied to gut microbiome datasets, successfully distinguishing CRC patients from healthy individuals with high accuracy (Teixeira et al., 2024). Deep learning models that integrate host transcriptomic and microbial features have further improved early cancer detection, particularly in malignancies such as pancreatic and lung cancer. Studies have shown that the incorporation of microbiome data into predictive models enhances the performance of traditional cancer screening methods, providing a more comprehensive assessment of disease status (Zheng et al., 2021).

The application of machine learning in microbiome-based cancer detection holds great promise for clinical implementation, offering a scalable and high-throughput approach to non-invasive screening and personalized oncology. The microbiome represents a valuable diagnostic and prognostic biomarker for various cancers, with microbial signatures in feces, saliva, and blood providing critical insights into disease detection and progression. The emergence of circulating microbial DNA as a liquid biopsy tool has further expanded the potential of microbiome-based diagnostics, offering a non-invasive alternative to traditional biopsy techniques. Moreover, the integration of machine learning with microbiome sequencing data has significantly improved cancer detection accuracy, paving the way for microbiome-driven precision oncology. Future research should focus on standardizing microbiome-based diagnostic assays and validating their clinical utility in large-scale patient cohorts, ultimately enhancing early detection strategies and improving cancer outcomes.

## 7 Influence of microbiota on cancer therapies

**FIGURE 3:**
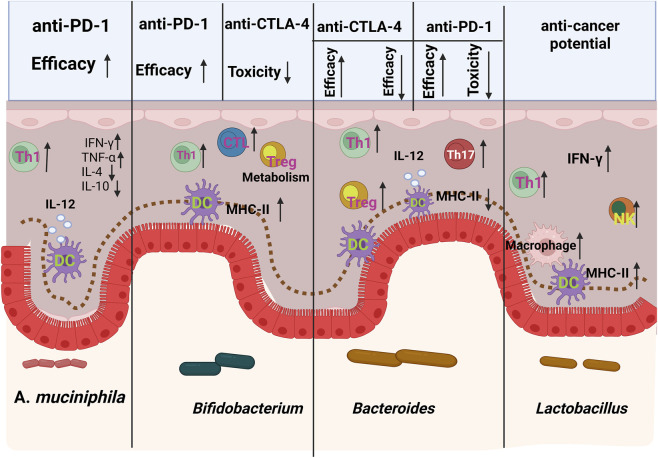
Influence of gut microbiota on cancer immunotherapy efficacy and toxicity. Distinct gut microbial taxa differentially modulate the efficacy and toxicity of cancer immunotherapies through immune priming, cytokine signaling, and antigen presentation. *Akkermansia muciniphila* (*A*. *muciniphila)* enhances anti-PD-1 therapy efficacy by promoting dendritic cell (DC) maturation, increasing interleukin-12 (IL-12) production, and driving Th1 polarization with elevated IFN-γ and TNF-α levels, leading to improved CD8^+^ cytotoxic T lymphocyte (CTL) responses. *Bifidobacterium* spp. support anti-PD-1 efficacy by enhancing DC antigen presentation (MHC-II), metabolic fitness of immune cells, and CTL activation while limiting regulatory T cell (Treg)–mediated immune suppression. *Bacteroides* spp. modulate anti-CTLA-4 responses by balancing Th1 and Th17 immune pathways, enhancing IL-12 signaling, and reducing immune-related toxicity associated with CTLA-4 blockade. *Lactobacillus* spp. contributes to reduced therapy-associated toxicity and improved anti-cancer potential by promoting innate immune activation, including natural killer (NK) cell function, macrophage activation, and IFN-γ production, while supporting antigen presentation by DCs. Collectively, these microbiota-driven immune mechanisms shape treatment responsiveness, therapeutic efficacy, and toxicity outcomes in cancer patients receiving immune checkpoint inhibitors.

The human microbiome has emerged as a critical determinant of cancer therapy outcomes, influencing both treatment efficacy and toxicity. Recent research has demonstrated that gut microbiota plays an essential role in shaping patient responses to checkpoint inhibitors, chemotherapy, and radiotherapy, largely through its effects on immune modulation, drug metabolism, and systemic inflammation. The microbiota can either enhance therapeutic efficacy by priming the immune system or contribute to treatment resistance through metabolic inactivation of drugs and immune suppression. Conversely, disruptions to microbiome composition, particularly through antibiotic use, have been associated with impaired immunotherapy responses and increased chemotherapy toxicity. This section explores the mechanisms by which gut microbiota enhances or hinders cancer therapy efficacy, highlighting its potential as a therapeutic target for improving treatment outcomes. The influence of gut microbiota on therapy is illustrated in Figure 3.

### 7.1 Microbiota-driven response to cancer treatments: Gut microbiota enhancing checkpoint inhibitor efficacy

Immune checkpoint inhibitors (ICIs) have revolutionized cancer treatment by enhancing T cell-mediated anti-tumor immunity, yet their effectiveness varies widely among patients. Emerging evidence suggests that gut microbiota composition plays a pivotal role in determining patient responsiveness to ICIs, with specific microbial taxa associated with improved treatment outcomes. Studies have identified *A*. *muciniphila*, *Bifidobacterium spp.*, and *Ruminococcaceae* as beneficial bacterial taxa that enhance PD-1 and CTLA-4 blockade efficacy. These bacteria contribute to T cell priming, activation, and infiltration into the tumor microenvironment, thereby enhancing immune responses (Xiaoyu et al., 2020). *A. muciniphila* has been shown to promote dendritic cell maturation and increase IL-12 production, leading to the recruitment and activation of CD8^+^ T cells, which are essential for effective tumor clearance (Chen et al., 2020; Shi et al., 2020). Furthermore, fecal microbiota transplantation (FMT) from immune checkpoint therapy responders to non-responding patients has successfully converted non-responders into responders, further confirming the role of the gut microbiota in shaping immunotherapy success (Davar et al., 2021). These findings highlight the potential for microbiota-targeted interventions, such as probiotics, prebiotics, and FMT, to enhance immune checkpoint blockade efficacy and improve patient outcomes.

### 7.2 Microbial metabolites reducing chemotherapy toxicity

In addition to influencing immunotherapy, gut microbiota plays a crucial role in modulating chemotherapy-induced toxicity. Chemotherapeutic agents often cause significant damage to the intestinal epithelium, leading to side effects such as diarrhea, mucositis, and systemic inflammation. However, microbial metabolites, particularly short-chain fatty acids (SCFAs) such as butyrate and propionate, have been shown to exert protective effects by promoting epithelial regeneration and reducing inflammation. SCFAs enhance gut barrier integrity by upregulating tight junction proteins, thereby reducing intestinal permeability and preventing chemotherapy-induced mucosal damage (Sadeghloo and Sadeghi, 2025). Additionally, butyrate has been shown to modulate immune responses by reducing pro-inflammatory cytokine production and promoting the expansion of regulatory T cells (Tregs), which mitigate chemotherapy-induced inflammation (Sun et al., 2024).

Furthermore, specific gut bacteria such as *Lactobacillus spp*. and *Bifidobacterium spp*. produce metabolites that neutralize oxidative stress and inflammation induced by chemotherapeutic agents, reducing off-target toxicity while maintaining anti-tumor efficacy (Khorashadizadeh et al., 2024). These findings suggest that modulating microbiota-derived metabolites through dietary interventions or probiotic supplementation could help mitigate chemotherapy side effects, improving patient quality of life.

### 7.3 Gut dysbiosis and treatment resistance: antibiotic use disrupting immunotherapy responses

While gut microbiota can enhance immunotherapy efficacy, disruptions to its composition, particularly through antibiotic use have been shown to impair immune checkpoint blockade responses. Broad-spectrum antibiotics can lead to gut dysbiosis, reducing the abundance of beneficial microbial taxa that are essential for T cell priming and activation.

Clinical studies have demonstrated that patients receiving antibiotics before or during ICI therapy exhibit significantly lower response rates and reduced overall survival (Elkrief et al., 2025). This effect is attributed to the depletion of *A*. *muciniphila* and *Bifidobacterium*, which play key roles in enhancing anti-tumor immunity. Moreover, antibiotic-induced dysbiosis is associated with an increase in pro-inflammatory and immunosuppressive bacterial species, which can lead to systemic inflammation and immune dysfunction (Dahiya and Nigam, 2023).

These findings suggest that preserving gut microbiota diversity is critical for optimizing immunotherapy responses. Strategies such as microbiota-based interventions, including FMT and probiotic supplementation, may help restore microbial diversity and improve treatment efficacy in patients who have undergone antibiotic exposure.

### 7.4 Microbiota-derived metabolites neutralizing chemotherapy agents

In addition to modulating chemotherapy toxicity, the gut microbiota can contribute to chemotherapy resistance through microbial metabolism of anti-cancer drugs. Certain bacterial species harbor enzymatic pathways that metabolize and inactivate chemotherapeutic agents, reducing their cytotoxic effects and diminishing treatment efficacy. One of the most well-documented examples is the bacterial degradation of 5-fluorouracil (5-FU), a widely used chemotherapeutic drug for colorectal, breast, and gastric cancers. Studies have identified that *Bacteroides* and *Enterococcus* species encode pyrimidine metabolism enzymes, which degrade 5-FU into inactive metabolites, thereby reducing drug availability and contributing to chemoresistance (Spanogiannopoulos et al., 2022). Similarly, bacterial β-glucuronidases present in the gut microbiota have been implicated in the reduction of irinotecan efficacy by reactivating its inactive glucuronide metabolite into a toxic form, leading to gastrointestinal toxicity and treatment discontinuation (Showe et al., 2009).

These findings highlight the need for microbiome-targeted strategies to counteract microbial inactivation of chemotherapy agents. Approaches such as selective antibiotic regimens, dietary modifications, and microbial enzyme inhibitors could help enhance chemotherapy efficacy and reduce the risk of treatment failure. The gut microbiota plays a pivotal role in shaping cancer therapy responses, influencing immune checkpoint blockade efficacy, chemotherapy toxicity, and treatment resistance. While beneficial microbial taxa can enhance therapy outcomes, gut dysbiosis and microbial metabolism of chemotherapeutic agents can contribute to treatment resistance and toxicity. Understanding these interactions provides opportunities for microbiota-targeted interventions, including FMT, probiotics, and microbial enzyme inhibitors, to optimize cancer therapy and improve patient survival.

## 8 Microbiome-based therapeutic strategies

### 8.1 Role of beneficial bacteria (*Lactobacillus*, Bifidobacterium) in reducing chemotherapy-induced toxicity

#### 8.1.1 Probiotics, prebiotics, and synbiotics

Chemotherapy-induced toxicity, particularly gastrointestinal toxicity, mucositis, and dysbiosis, remains a major challenge in cancer treatment. The gut microbiota plays a key role in modulating chemotherapy responses, and disruptions in microbial composition can exacerbate intestinal inflammation, increase gut permeability, and compromise immune homeostasis. Probiotics, particularly species from the *Lactobacillus* and *Bifidobacterium* genera, have shown promising effects in alleviating chemotherapy-associated toxicities by reinforcing gut barrier integrity, reducing inflammation, and restoring microbial balance (Akbarali et al., 2022). *Lactobacillus* species, such as *L*. *rhamnosus GG* and *Lactobacillus casei*, have been widely studied for their protective effects against chemotherapy-induced mucositis.

These probiotics help maintain tight junction integrity in intestinal epithelial cells, preventing bacterial translocation and subsequent systemic inflammation. Additionally, *L*. *rhamnosus* GG supplementation has been shown to reduce the severity of diarrhea and mucosal damage in cancer patients undergoing fluoropyrimidine and platinum-based chemotherapy regimens (Ciorba et al., 2015). Similarly, *Bifidobacterium* species, such as *B*. *breve* and *B*. *longum*, have demonstrated anti-inflammatory effects that mitigate gut inflammation and oxidative stress induced by chemotherapy. These bacteria produce SCFAs, particularly butyrate, which serve as an energy source for colonocytes and promote intestinal epithelial repair. In clinical trials, supplementation with Bifidobacterium breve was associated with reduced gut permeability and lower levels of inflammatory cytokines, suggesting a potential role in ameliorating chemotherapy-induced gastrointestinal toxicity (Sharma et al., 2024).

Beyond gut protection, probiotic administration has been linked to improved chemotherapy tolerance and immune modulation. Studies have shown that probiotics modulate the gut microbiota composition to favor anti-inflammatory bacteria, thereby preventing dysbiosis-related complications such as opportunistic infections and secondary gut-derived inflammation (López-Gómez et al., 2023). These findings underscore the importance of incorporating probiotic-based strategies to enhance chemotherapy tolerability and reduce treatment-related complications. The clinical relevance of different interventions on cancer is documented in Table 4.

**TABLE 4:** Clinical relevance of different therapeutic interventions in different cancer types.

Therapeutic strategy	Intervention type	Key microbes/Metabolites	Cancer type	Primary mechanisms	Clinical relevance in cancer therapy	Key references (hyperlinked DOI)
Probiotics	Live beneficial bacteria	*B*. *longum, L*. *lactis, and E*. *faecium*	Advanced nasopharyngeal carcinoma	Reduced systemic inflammation and protects mucosa	Immune System Support, Intestinal Microbiota Restoration, Reduced GI Toxicity and Inflammation	Jiang et al. (2019)
Probiotics	Live beneficial bacteria	*B*. *breve*, *B. longum*	Breast Cancer	SCFA (butyrate) production, anti-inflammatory effects, epithelial repair	Mitigates chemotherapy-induced gut permeability and cytokine release	Ahmad et al. (2025)
Probiotics (immune modulation)	Live beneficial bacteria	*Lactobacillus*, *Bifidobacterium* spp.	Colorectal Cancer	Restoration of microbial balance, prevention of dysbiosis-related inflammation	Improves chemotherapy tolerance and reduces opportunistic infections	Petrariu et al. (2024)
Prebiotics	Non-digestible dietary fibers	Inulin, FOS, GOS, resistant starch	Colorectal Cancer	Selective enrichment of beneficial microbes, SCFA production	Restores microbial diversity and gut homeostasis after cancer therapy	Vassiliou et al. (2023)
Prebiotics (immunotherapy support)	Dietary modulation	Fiber-induced SCFAs	Melanoma, Non-Small Cell Lung Cancer	Enhanced GALT activity, increased CD8^+^ T-cell responses	Improves response to PD-1/PD-L1 immune checkpoint inhibitors	Zheng et al. (2025)
Prebiotics (dysbiosis control)	Dietary supplementation	SCFA-producing taxa	Gastrointestinal Cancers (especially Colorectal Cancer)	Suppression of *Enterobacteriaceae*, reduced inflammation	Reduces chemotherapy-associated GI toxicity	Sifrim et al. (2007)
Synbiotics	Probiotics + prebiotics	*Bifidobacterium* + inulin	Colorectal Cancer	Synergistic enhancement of microbial stability and gut barrier	Superior gut protection compared to probiotics alone	Smolinska et al. (2025)
FMT	Microbiota transfer	Donor-derived diverse microbiota	Melanoma, Lung Cancer	Restoration of microbial diversity, immune modulation	Improves dysbiosis and treatment tolerance	Routy et al. (2018)
FMT (Immunotherapy)	Microbiota transfer	*A.muciniphila*, *Bifidobacterium* spp.	Metastatic Melanoma	Enhanced antigen presentation, CD8^+^ T-cell activation	Converts immunotherapy non-responders to responders	Matson et al. (2018)
FMT – Challenges	Clinical/ethical considerations	​	Multiple Cancer Types	Risk of pathogen transfer, lack of standardization	Limits widespread clinical adoption	Yadegar et al. (2024)
High-fiber diet	Dietary intervention	SCFA-producing bacteria	Melanoma	Increased butyrate, reduced inflammation, immune modulation	Enhances immunotherapy efficacy and reduces toxicity	Xiong et al. (2022)
Postbiotics	Microbial metabolites	Butyrate, propionate	Colorectal Cancer	Treg induction, suppression of inflammatory cytokines	Precision immunomodulation without live bacteria	Tang et al. (2011)
Microbial metabolites	Polyamines	Spermidine	Colorectal Cancer, Liver Cancer	Anti-inflammatory, anti-aging, immune regulation	Potential adjunct therapy in cancer and aging	Chenna et al. (2025)
Microbiome engineering	Genetically modified bacteria	*Clostridium*, *Salmonella* spp.	Solid Tumors (e.g., Colorectal, Pancreatic, Melanoma)	Tumor colonization, local drug delivery	Precision tumor targeting	Varsha and Dwidar (2026)
CRISPR-based modulation	Genome editing	Engineered gut bacteria	Multiple Cancer Types	Selective elimination or enhancement of taxa	Personalized microbiome-based cancer therapy	Thuronyi et al. (2019)

#### 8.1.2 Prebiotic dietary interventions enhancing gut microbiome diversity

Prebiotics are non-digestible dietary fibers that selectively stimulate the growth of beneficial gut bacteria, thereby improving microbiome diversity and resilience. Given that gut dysbiosis is a common consequence of cancer therapies, prebiotic interventions have been explored as a means to restore microbial balance, enhance immune function, and improve metabolic homeostasis (Ji et al., 2023). Several prebiotic compounds, including fructooligosaccharides (FOS), inulin, and galactooligosaccharides (GOS), have been shown to enhance the proliferation of *Lactobacillus* and *Bifidobacterium* species, leading to improved gut health and immune modulation. In cancer patients, dietary supplementation with inulin and resistant starch has been associated with increased microbial diversity and higher levels of SCFA production, which contribute to gut epithelial integrity and immune homeostasis (Bruno-Barcena and Azcarate-Peril, 2015).

Additionally, prebiotic-rich diets have been found to modulate gut-associated lymphoid tissue (GALT) activity, leading to enhanced mucosal immunity and a reduction in pro-inflammatory cytokine levels. This effect is particularly relevant in patients undergoing immune checkpoint blockade therapy, where prebiotic consumption has been associated with increased levels of CD8^+^ T cells and improved response rates to anti-PD-1/PD-L1 therapy (Hardy et al., 2013; Wu et al., 2017). Beyond immune modulation, prebiotics may also mitigate chemotherapy-induced dysbiosis by preventing the overgrowth of pathogenic bacteria that contribute to gastrointestinal toxicity. Studies have shown that prebiotic supplementation reduces the abundance of *Enterobacteriaceae* and other inflammation-promoting taxa, while promoting the growth of SCFA-producing bacteria that exert protective effects on the gut lining (Khorashadizadeh et al., 2024).

In clinical applications, combining prebiotics with probiotics (synbiotics) has demonstrated synergistic benefits, further enhancing gut microbiota diversity and metabolic function. Studies have reported that synbiotic formulations containing *Bifidobacterium* and inulin lead to greater microbial stability and improved gut barrier function, compared to probiotics or prebiotics alone (Smolinska et al., 2025). These findings highlight the potential of prebiotic interventions in cancer therapy, particularly for reducing treatment-related dysbiosis and improving microbiome resilience.

#### 8.1.3 Fecal microbiota transplantation (FMT)

Fecal microbiota transplantation (FMT) has emerged as a promising therapeutic strategy for restoring gut microbial diversity, with significant implications for improving responses to cancer therapies, particularly immune checkpoint inhibitors (ICIs). By transferring stool-derived microbiota from healthy donors to patients experiencing gut dysbiosis, FMT has been shown to replenish beneficial microbial taxa, modulate immune responses, and reduce inflammation. Initially established for treating recurrent Clostridioides difficile infections, FMT is now being actively explored in oncology to enhance treatment efficacy and mitigate therapy-related side effects.

### 8.2 FMT in immunotherapy: Restoring microbiome diversity to improve checkpoint inhibitor responses

The gut microbiota plays a pivotal role in shaping the host immune system, influencing responses to immune checkpoint blockade therapy. Patients who respond favorably to PD-1 and CTLA-4 inhibitors exhibit distinct microbiome signatures enriched in beneficial bacterial taxa, such as *A*. *muciniphila* and *Bifidobacterium* spp., which are associated with enhanced T cell activation and tumor clearance (H. Jiang and Zhang, 2024; Routy et al., 2018). FMT has been investigated as a potential method to convert non-responders into responders by modifying the gut microbiota composition.

In a landmark study, FMT from immunotherapy responders to non-responders successfully restored anti-tumor immune responses, leading to increased infiltration of CD8^+^ T cells into tumors and improved therapeutic efficacy (Davar et al., 2021; Ramesh et al., 2026). These findings underscore the critical role of the gut microbiome in immunotherapy outcomes, suggesting that microbiome modulation through FMT may enhance patient responses to checkpoint blockade therapy. Mechanistically, FMT is believed to improve dendritic cell function, promote antigen presentation, and stimulate the production of pro-inflammatory cytokines such as IL-12, which further boosts T cell activation (Matson et al., 2018). Given these immunomodulatory effects, FMT represents a novel strategy for optimizing cancer immunotherapy.

#### 8.2.1 Challenges and ethical considerations in clinical applications

Despite its potential, the clinical implementation of FMT in oncology is associated with several challenges and ethical concerns. One of the primary limitations is safety and donor selection, as FMT carries the risk of transmitting opportunistic pathogens or drug-resistant bacteria. Stringent donor screening protocols are essential to minimize the risk of infections and ensure patient safety (Ramesh et al., 2026; Wardill et al., 2019). Furthermore, the lack of standardization in FMT protocols, including variations in microbiota preparation, storage, and delivery methods, poses a significant barrier to clinical translation. Current research efforts are focused on developing synthetic microbiota formulations, such as defined consortia of beneficial bacterial strains, which may offer a more controlled and reproducible alternative to FMT (Mullish et al., 2018; Scarpellini et al., 2025). Ethical considerations also arise regarding patient consent and accessibility, as FMT remains an experimental therapy in oncology. Given the complexities of regulatory approval, patient safety, and clinical efficacy, further research is needed to establish clear guidelines for FMT use in cancer treatment.

### 8.3 Dietary and metabolite-based interventions

Dietary interventions have a profound impact on gut microbiome composition, influencing immune function, metabolic pathways, and therapeutic responses. Recent studies suggest that specific dietary components, including high-fiber foods and microbial metabolites, can enhance treatment efficacy and reduce side effects associated with chemotherapy and immunotherapy. Furthermore, postbiotics bioactive metabolites produced by gut microbes are being investigated as precision therapeutics for cancer therapy.

#### 8.3.1 High-fiber diets enhancing beneficial gut microbial populations

A high-fiber diet is associated with increased microbial diversity and the expansion of beneficial SCFA-producing bacteria, which exert anti-inflammatory and immunomodulatory effects. Dietary fiber fermentation by gut bacteria generates butyrate, propionate, and acetate, which reinforce gut barrier integrity, reduce inflammation, and modulate host immune responses (Meiners et al., 2025). In cancer patients, high-fiber intake has been correlated with enhanced responses to PD-1 inhibitors, possibly due to increased microbial diversity and improved gut-immune interactions. Additionally, high-fiber diets have been linked to reduced systemic inflammation and lower rates of treatment-associated gut toxicity, further supporting their role in cancer therapy (Gamrath et al., 2025).

#### 8.3.2 Postbiotics and microbial metabolites as precision therapeutics

Postbiotics, including SCFAs, bacterial lysates, and metabolic byproducts, offer an alternative to probiotics by delivering bioactive compounds directly without requiring live bacterial colonization. SCFAs, particularly butyrate and propionate, have been shown to enhance regulatory T cell function, reduce inflammatory cytokine production, and improve anti-tumor immune responses (Pattapulavar et al., 2025). Additionally, emerging research suggests that microbial-derived polyamines, such as spermidine, exhibit anti-aging and anti-inflammatory properties, which may have therapeutic potential in modulating tumor progression and immune responses (Holbert et al., 2022). Future clinical studies should focus on optimizing metabolite-based therapies to improve cancer treatment outcomes.

### 8.4 Microbiome engineering and synthetic biology

Recent advancements in synthetic biology and microbiome engineering have enabled the development of genetically modified bacteria for tumor targeting and drug delivery. By engineering bacterial strains to colonize tumors, release therapeutic agents, and modulate host immunity, researchers are exploring innovative strategies for precision oncology. Additionally, CRISPR-based genome editing technologies are being applied to selectively modulate the microbiome, allowing for the precise elimination of pathogenic species or the introduction of beneficial bacteria.

#### 8.4.1 Genetically modified bacteria for tumor targeting and drug delivery

Certain anaerobic bacteria, including *Clostridium* and *Salmonella* species, naturally accumulate within hypoxic tumor microenvironments, making them ideal candidates for microbial-based cancer therapy. Engineered bacterial strains have been developed to secrete tumoricidal compounds, stimulate anti-tumor immune responses, and improve drug delivery (Aganja et al., 2022).

#### 8.4.2 CRISPR-based approaches for microbiome modulation

CRISPR-based technologies have provided a powerful tool for precise microbiome modulation, allowing researchers to eliminate harmful bacterial strains or enhance beneficial microbes. CRISPR-Cas9 has been successfully applied to engineer gut bacteria for improved immune modulation and drug metabolism, offering a new Frontier in microbiome-based cancer therapies (Amen et al., 2025). Future research should focus on optimizing CRISPR-based microbiome engineering for personalized therapeutic interventions in oncology.

## 9 Challenges and future directions

### 9.1 Interindividual variability and methodological standardization

As research on the microbiome-cancer interface expands, there is increasing recognition of its potential in cancer diagnostics, prognostics, and therapeutics. Despite promising advances, several challenges hinder the clinical translation of microbiome-based interventions, including variability in microbiome composition, lack of methodological standardization, regulatory hurdles, and safety concerns.

One of the foremost challenges in microbiome research is the high inter-individual variability in gut microbial composition, which makes it difficult to develop universal microbiome-based biomarkers or therapies. Several factors, including diet, genetics, geographic location, ethnicity, medication exposure, and prior medical history, influence microbiome composition, leading to inconsistencies in study outcomes. This variability complicates the identification of reproducible microbial signatures for cancer detection and treatment response prediction (Yao et al., 2026).

Another critical limitation is the lack of standardized methodologies in microbiome research. Differences in sample collection, DNA extraction protocols, sequencing techniques, and bioinformatics pipelines can significantly impact study reproducibility. The use of various sequencing technologies, such as 16S rRNA gene sequencing, shotgun metagenomics, and metatranscriptomics, introduces discrepancies in microbiome datasets, making it challenging to compare findings across studies (Ece et al., 2026). Developing standardized protocols and reference datasets will be essential to ensure data reliability and facilitate cross-study comparisons.

### 9.2 Distinguishing correlation from causation

A fundamental challenge in microbiome-oncology research is distinguishing causal microbial drivers from taxa that are merely associated with disease states. While microorganisms such as *Helicobacter pylori* have well-established causal roles in carcinogenesis, many microbial signatures identified through sequencing studies remain largely correlative. Determining whether specific microbes initiate tumor development, promote tumor progression, or simply thrive within tumor-associated environments remains an unresolved question. Addressing this challenge will require longitudinal cohort studies, mechanistic validation in experimental models, and integration of microbial, host, and environmental data.

Additionally, conflicting findings are frequently reported across microbiome studies. Microbial taxa associated with cancer risk, prognosis, or treatment response in one cohort are not always reproducible in independent populations. Differences in study design, patient demographics, environmental exposures, sequencing platforms, and analytical pipelines contribute to these inconsistencies. Consequently, caution is warranted when interpreting microbiome signatures as universal biomarkers, and greater emphasis should be placed on reproducibility and external validation.

### 9.3 Regulatory and safety challenges

Another major concern is the regulatory and safety considerations surrounding microbiome-based interventions. Fecal microbiota transplantation (FMT), for example, remains unapproved by regulatory agencies for cancer therapy, largely due to concerns regarding pathogen transmission, donor variability, and patient safety (Grigoryan et al., 2020). Similarly, probiotic and prebiotic interventions lack consistent regulatory oversight, with varying quality control standards across commercially available formulations. Establishing clear regulatory frameworks and safety guidelines will be critical for the clinical implementation of microbiome-targeted therapies.

Although encouraging responses have been reported in selected patients and early clinical studies, treatment outcomes remain highly variable and may depend on donor microbiota composition, recipient characteristics, tumor type, and concomitant therapies. Consequently, larger randomized clinical trials are required before FMT can be routinely incorporated into cancer treatment strategies.

### 9.4 Unresolved mechanistic questions

Despite significant advances in understanding microbiome-cancer interactions, several mechanistic questions remain unresolved. The context-dependent effects of microbial metabolites, including short-chain fatty acids, the precise contribution of tumor-associated microbiota to local immune suppression, and the complex interplay between microbial signaling, host epigenetic regulation, and immune checkpoint responses are not yet fully understood. Furthermore, it remains unclear whether many tumor-associated microbes function as primary drivers of oncogenesis, facilitators of tumor progression, or opportunistic colonizers of established tumors. Elucidating these mechanisms will be essential for translating microbiome discoveries into clinically actionable interventions.

Taken together, current evidence suggests that the microbiome should not be viewed as a single universal determinant of cancer risk or therapeutic response. Rather, microbiome-cancer interactions are highly context-dependent and influenced by host genetics, environmental exposures, dietary factors, tumor type, and treatment history. Future studies must move beyond cataloging microbial associations and focus on establishing causal mechanisms, validating reproducible biomarkers, and defining clinically actionable microbiome signatures.

### 9.5 Future perspectives in precision oncology

The future of microbiome-based cancer research lies in precision medicine approaches that integrate multi-omics data, artificial intelligence (AI), and personalized microbiome interventions. Advances in host-microbiome interaction studies using metagenomics, metabolomics, and transcriptomics will allow researchers to identify individualized microbial signatures that can guide personalized cancer therapies. AI-driven models are being developed to analyze microbiome data in combination with clinical parameters, potentially improving early cancer detection and treatment prediction (Azhdarimoghaddam et al., 2025).

Emerging fields such as synthetic biology and microbiome engineering hold great promise for developing genetically modified bacterial strains capable of delivering targeted therapeutics within the tumor microenvironment. CRISPR-based approaches could allow for precise manipulation of gut microbiota, enabling researchers to selectively enhance beneficial bacterial species while suppressing harmful microbes (Cheng et al., 2025). Additionally, next-generation probiotics and postbiotics are being developed to modulate immune responses and improve therapy outcomes. Unlike traditional probiotics, these advanced formulations may include engineered bacterial strains with enhanced metabolic capabilities that can augment chemotherapy efficacy, reduce inflammation, and improve gut barrier integrity (Abouelela and Helmy, 2024). However, most of these approaches remain in preclinical or early clinical stages and require rigorous validation before routine clinical implementation.

Large-scale clinical trials and longitudinal studies will be necessary to validate microbiome-targeted interventions and assess their long-term safety and efficacy. Future studies should focus on understanding host-microbiome interactions, optimizing microbiome modulation strategies, and translating basic research into clinically viable therapies.

## 10 Conclusion

The microbiome has emerged as a key factor in cancer biology, influencing tumor development, disease progression, and responses to therapy. Advances in sequencing technologies, metagenomics, and systems biology have improved our understanding of how microbial communities interact with the host and shape cancer-related processes. These discoveries have highlighted the microbiome’s potential role in improving cancer prevention, diagnosis, and treatment. Microbiome-based interventions such as probiotics, prebiotics, fecal microbiota transplantation (FMT), and dietary modifications are being explored as strategies to enhance treatment effectiveness and reduce therapy-related side effects. Research has shown that gut microbiota composition can influence the success of cancer immunotherapy, including immune checkpoint inhibitors. Modulating microbiomes may therefore strengthen anti-tumor immune responses and improve patient outcomes. The microbiome is also gaining attention as a potential source of non-invasive diagnostic biomarkers. Techniques such as fecal microbiome profiling, salivary microbiota analysis, and detection of circulating microbial DNA are being investigated for early cancer detection and prognosis. When combined with machine learning and multi-omics analysis, these methods may enable more accurate and personalized cancer screening approaches. However, the complexity and variability of the human microbiome present challenges in identifying reliable and universally applicable biomarkers. Future research should focus on large-scale clinical trials and longitudinal studies to validate microbiome-based diagnostic and therapeutic strategies. Emerging technologies such as engineered bacteria, microbiome-derived metabolites, and synthetic biology approaches may offer more targeted and precise interventions. Integrating microbiology, oncology, and artificial intelligence will be essential to translate microbiome discoveries into effective tools for precision cancer medicine.
